# High-throughput total RNA sequencing in single cells using VASA-seq

**DOI:** 10.1038/s41587-022-01361-8

**Published:** 2022-06-27

**Authors:** Fredrik Salmen, Joachim De Jonghe, Tomasz S. Kaminski, Anna Alemany, Guillermo E. Parada, Joe Verity-Legg, Ayaka Yanagida, Timo N. Kohler, Nicholas Battich, Floris van den Brekel, Anna L. Ellermann, Alfonso Martinez Arias, Jennifer Nichols, Martin Hemberg, Florian Hollfelder, Alexander van Oudenaarden

**Affiliations:** 1 Hubrecht Institute-KNAW (Royal Netherlands Academy of Arts and Sciences) and University Medical Center, Utrecht, Netherlands; 2 Oncode Institute, Utrecht, Netherlands; 3 Department of Biochemistry, University of Cambridge, Cambridge, UK; 4 Wellcome Sanger Institute, Wellcome Genome Campus, Hinxton, UK; 5 Division of Stem Cell Therapy, Center for Stem Cell Biology and Regenerative Medicine, Institute of Medical Science, University of Tokyo, Tokyo, Japan; 6 Wellcome Trust – Medical Research Council Stem Cell Institute, University of Cambridge, Jeffrey Cheah Biomedical Centre, Cambridge, UK; 7 Systems Bioengineering, DCEXS, Universidad Pompeu Fabra, Doctor Aiguader 88 ICREA (Institució Catalana de Recerca i Estudis Avançats), Barcelona, Spain; 8 Department of Physiology, Development and Neuroscience, University of Cambridge, Cambridge, UK; 9 Evergrande Center for Immunologic Diseases, Harvard Medical School and Brigham and Women’s Hospital, Boston, MA USA; 10 Present Address: Francis Crick Institute, London, UK; 11 Present Address: Department of Environmental Microbiology and Biotechnology, Institute of Microbiology, Faculty of Biology, University of Warsaw, Warsaw, Poland

**Keywords:** Gastrulation, Transcriptomics, Non-coding RNAs, RNA splicing, Databases

## Abstract

Most methods for single-cell transcriptome sequencing amplify the termini of polyadenylated transcripts, capturing only a small fraction of the total cellular transcriptome. This precludes the detection of many long non-coding, short non-coding and non-polyadenylated protein-coding transcripts and hinders alternative splicing analysis. We, therefore, developed VASA-seq to detect the total transcriptome in single cells, which is enabled by fragmenting and tailing all RNA molecules subsequent to cell lysis. The method is compatible with both plate-based formats and droplet microfluidics. We applied VASA-seq to more than 30,000 single cells in the developing mouse embryo during gastrulation and early organogenesis. Analyzing the dynamics of the total single-cell transcriptome, we discovered cell type markers, many based on non-coding RNA, and performed in vivo cell cycle analysis via detection of non-polyadenylated histone genes. RNA velocity characterization was improved, accurately retracing blood maturation trajectories. Moreover, our VASA-seq data provide a comprehensive analysis of alternative splicing during mammalian development, which highlighted substantial rearrangements during blood development and heart morphogenesis.

## Abstract (web summary)

VASA-seq sequences total RNA in single cells, revealing non-coding expression and splicing patterns.

## Main

Single-cell RNA sequencing (scRNA-seq) has transformed understanding of cellular complexity over the last decade. Initial technologies were applied to small numbers of individual cells^1–4^ and were subsequently adapted to droplet microfluidics to sample thousands to millions of single cells^5–7^. Although state-of-the-art scRNA-seq methods are sufficiently sensitive to quantify and determine cell states with high accuracy^8–11^, most methods rely on the hybridization of barcoded oligo-dT primers to the poly(A) sequences of polyadenylated transcripts for RNA capture and complementary DNA (cDNA) synthesis. This results in the detection of short fragments (~400–600 base pairs) immediately adjacent to the poly(A) tail or at the 5′ end of the transcript, and, thus, remaining sequences in polyadenylated RNA molecules and the spectrum of non-polyadenylated transcripts are undetected. This prevents differential expression of non-coding RNAs and alternative splicing (AS) and alternative promoter (AP) usage analyses.

Full-length transcriptome sequencing methods^12,13^ have enabled AS profiling of polyadenylated RNA species at single-cell resolution^10,14,15^, but the exact quantification of splicing events is hampered by the lack of strand and unique molecular identifier (UMI) information along the whole gene body. Furthermore, neither full-length nor whole-transcriptome methods^16–18^ have been adapted to high-throughput droplet-based platforms, which offer at least one order-of-magnitude gain in throughput compared to plate-based methods^19^.

To overcome these challenges, we developed ‘vast transcriptome analysis of single cells by dA-tailing’ (VASA-seq), which captures both non-polyadenylated and polyadenylated transcripts across their length in both plate and droplet microfluidic formats. We first benchmarked VASA-seq against state-of-the-art methods using cultured cells. To our knowledge, VASA-seq is the only technology to combine excellent sensitivity, full-length coverage of total RNA and high throughput. Next, we used VASA-seq to sample more than 30,000 single cells from mouse post-implantation embryos at the following developmental stages: embryonic day (E) 6.5, E7.5, E8.5 and E9.5. Our resource provides a comprehensive analysis of mammalian post-implantation development by characterizing the total transcriptome at single-cell resolution. The analysis revealed layers of biological information that have been absent from recently published resources^20–24^. Indeed, VASA-seq’s increased sensitivity led to the discovery of several cell-type-specific marker genes and non-polyadenylated histone gene expression patterns, which were used to accurately determine cell cycle stage across tissues. Higher coverage of intronic regions in the full-length VASA-seq dataset led to more accurate RNA velocity measurements^25,26^ across differentiation trajectories. Finally, we used the full-length coverage to determine cell-type-specific splicing patterns, with an emphasis on heart morphogenesis and blood development. Taken together, VASA-seq is a sensitive and scalable single-cell technology that uncovers a layer of biological information not attainable with technologies that rely on the current mRNA termini-centric view.

## Results

### VASA-seq enables detection of both non-polyadenylated and polyadenylated transcripts in single cells using plates or droplets

**Fig. 1:**
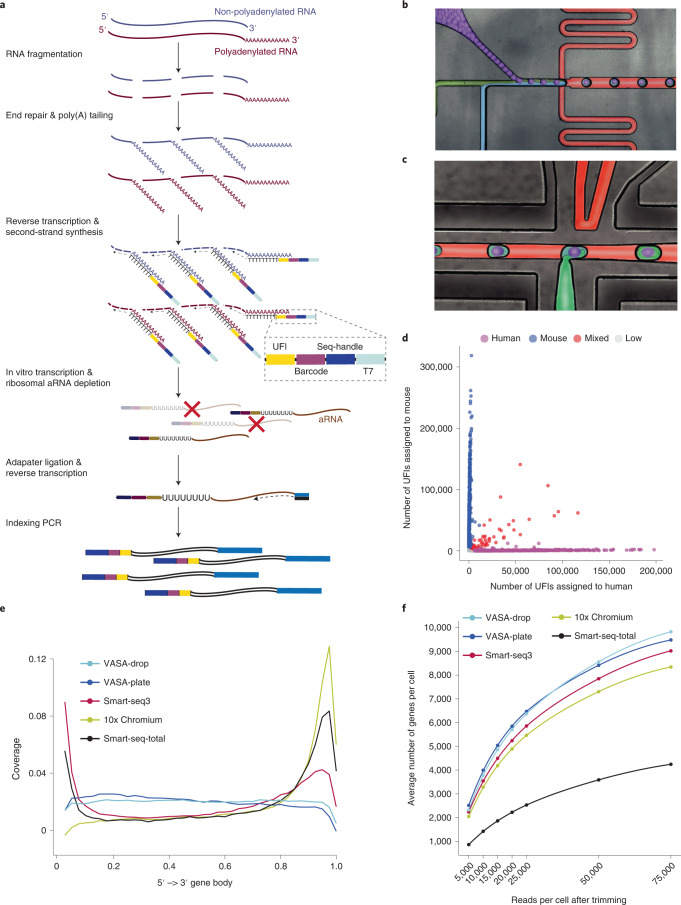
Overview of the VASA-seq workflow and benchmarking against other state-of-the-art methodologies. **a**, Overview of the VASA-seq single-cell molecular workflow. Single cells are lysed, and RNA is fragmented. Fragments are repaired and polyadenylated, followed by reverse transcription (RT) using barcoded oligo-dT primers. The cDNA is made double stranded and amplified using IVT. aRNA is depleted of rRNA, and libraries are finalized by ligation, RT and PCR, which leave fragments ready for sequencing. **b**, Picture illustrating the single-cell encapsulation process using droplet microfluidics. The single cells (green) are co-encapsulated with a barcoded bead (purple), lysis and fragmentation mix (blue), and compartmentalization is achieved with the addition of fluorinated surfactant oil (red) at the flow-focusing junction. **c**, Picture illustrating the picoinjection of reagents (green) to single-cell lysates (light blue/purple). The droplet surface tension is perturbed using an electric field that allows for the subsequent additions of end repair/poly(A) and RT mix. **d**, Cross-contamination test for VASA-drop was carried out using HEK293T cells (human) and mouse embryonic stem cells (mouse). Barcodes with more than 25% of detected UFIs belonging to the other species were considered doublets/mixed (red). Detected barcodes with low UFIs (<7,500) were discarded (gray). The remainder were assigned to either human (magenta) or mouse (blue). **e**, Gene body coverage comparison along protein-coding genes. VASA-seq showed even coverage, whereas 10x, Smart-seq-total and Smart-seq3 had a bias toward transcript termini (3′ or 5′ and 3′, respectively). **f**, The number of detected annotated genes in HEK293T cells, for each method, is plotted against the number of reads (after quality filtering, adapter removal and homopolymer trimming) per cell across different downsampling thresholds. The saturation curves showed that VASA-seq was the most sensitive of the methods. Curvature of gene detection indicated that full complexity was not reached for the method when 75,000 reads were allocated to each cell. Only cells that were sequenced to at least 75,000 reads were used (VASA-plate: *n* = 174, VASA-drop: *n* = 376, Smart-seq3: *n* = 113, Smart-seq-total = 260, 10x Chromium: *n* = 288).

**Extended Data Fig. 1:**
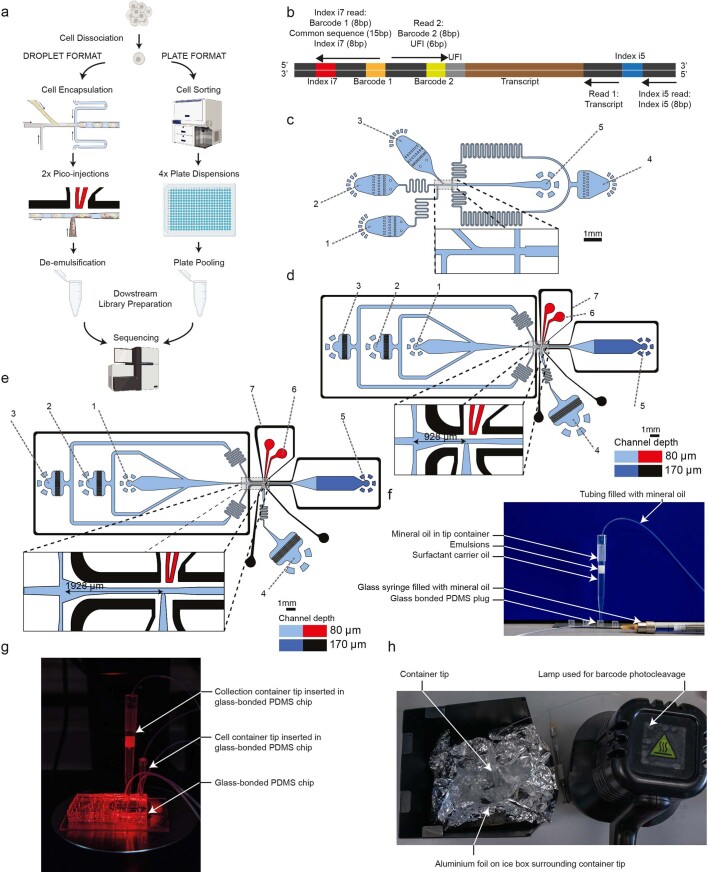
Overview of the sequencing and droplet microfluidic process and benchmarking analysis. **a**, The two platforms for VASA-seq, using a microfluidic device (left, VASA-drop) and a plate dispenser (right, VASA-plate). The microfluidic device allows the generation of single-cell libraries from thousands of cells, while the plate-based approach is better for rare cell types where a prior sorting is required. Each library contains the transcriptome from a large mix of cells, which are demultiplexed based on their barcode and index sequences. **b**, Library barcoding design for the VASA-drop workflow. Mouse embryo libraries were sequenced with the Illumina NovaSeq platform. To avoid index hopping, a custom dual indexing strategy was used. For the index i7 read, which usually only contains barcode 1 (inDrop v3), we inserted a 8-bp second index directly after a 15 bp common sequence. Only reads that had the correct combination of i5 and i7 index were further used for downstream processing. **c**, Design of the device used for barcoded bead and single-cell co-encapsulation. 1) input channel for the lysis and fragmentation mix, 2) input channel for the cell loading, 3) input channel for the barcoded compressible bead loading, 4) input channel for the fluorinated oil with admixed surfactant, 5) droplet exit channel. **d**, Design of the first picoinjector device, to inject the repair and poly(A) polymerase. 1) input channel for droplet reinjection, 2) emulsion diluting oil inlet, 3) droplet spacing oil inlet, 4) inlet for the repair and poly(A) polymerase to be picoinjected, 5) droplet exit channel, 6) positive electrode (red), 7) negative (moat) electrode (black). **e**, Design of the second picoinjector device, to inject the RT enzyme mix. 1) input channel for droplet re-injection, 2) emulsion diluting oil inlet, 3) droplet spacing oil inlet, 4) inlet for the RT mix to be picoinjected, 5) droplet exit channel, 6) positive electrode (red), 7) negative (moat) electrode (black). **f**, Photography of the container tip used for droplet collection and reinjection in the picoinjector devices. The tip is connected to a glass syringe which enables aspiration and delivery of emulsions. The tip can be connected to a PDMS plug to close the system for incubation in the water bath. **g**, Photography of the encapsulation process. The container tip collecting the emulsions is plugged into the outlet of the encapsulation device, while a tip containing cells is plugged in one of the inputs to deliver single-cells in the droplets. **h**, Photography of the bead barcode photocleavage set-up. The container tip is surrounded by aluminium foil to reflect the UV light and is kept on a container filled with ice.

The first step in the VASA-seq protocol entails the fragmentation of RNA molecules from the single-cell lysate followed by end repair and poly(A) tailing, enabling cDNA synthesis from barcoded oligo-dT probes. In addition, a unique fragment identifier (UFI) allows for the accurate quantification of molecules with strand specificity. Barcoded cDNA is amplified using in vitro transcription, and the amplified ribosomal RNA (rRNA) is subsequently depleted. The final stages of the protocol resemble the CEL-seq workflow^1^ (Fig. 1a and Extended Data Fig. 1a). Libraries are amplified using unique dual-indexed polymerase chain reaction (PCR) primers to enable the detection of index hopping when using the Illumina NovaSeq platform (Extended Data Fig. 1b).

We adapted the VASA-seq workflow to both plate (VASA-plate) and droplet microfluidic (VASA-drop) formats (Extended Data Fig. 1a,c–e). The plate-based format is widely available and can be set up with a variety of different robots made for plate dispensation at the nanoliter scale. Plates are also beneficial when dealing with smaller numbers of rare cell types and/or when cell sorting is required. The VASA-plate workflow works by sorting cells into plates containing primers and oil^27^, followed by consecutive reagent dispensing (Extended Data Fig. 1a). On the other hand, VASA-drop can be used for large-scale characterization of cell populations with less hands-on time and lower reagent costs. For this workflow, three microfluidic chip devices were optimized to run the reactions at high throughput. First, a modified flow-focusing device, similar to the inDrop workflow^5^, is used to co-compartmentalize cells, compressible barcoded polyacrylamide beads and a lysis/fragmentation buffer in sub-nanoliter water-in-oil emulsions (Fig. 1b, Extended Data Fig. 1c,f–h and Supplementary Video 1). The cell/bead co-encapsulation rate was calculated as 86% (based on analysis of video recordings; Supplementary Table 1). Co-encapsulation is followed by the addition of end-repair/poly(A) tailing and RT mixes in two consecutive steps of high-throughput reagent injections into each droplet using picoinjections^28^ (Fig. 1c, Extended Data Fig. 1d,e and Supplementary Video 2), with an estimated success rate of 98% per picoinjection (estimated from video recordings; Supplementary Table 1). The droplets are then de-emulsified and processed for downstream library preparation.

### Barcode mixing, biotype detection, gene body coverage and sensitivity of VASA-seq

**Extended Data Fig. 2:**
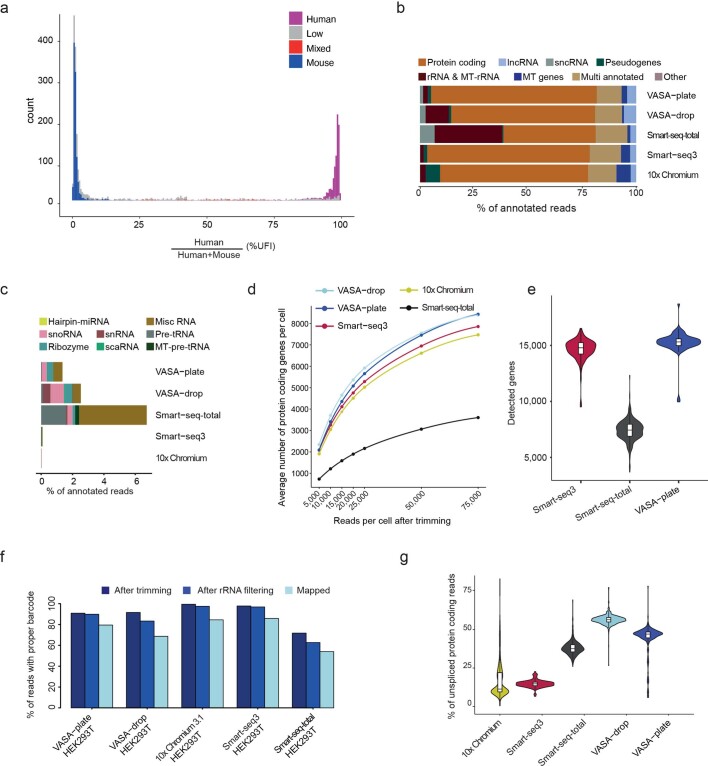
Comparison to other methods. **a**, Species mixing histogram plotted as a percentage of UFIs quantified from mapping events to a human reference genome, divided by the sum of UFIs quantified from mapping events to mouse and human reference genomes. **b**, Proportion of mapped reads to all annotated genes for each biotype using HEK293T cells across all methods. VASA-seq detected proportionally larger amounts of lncRNA genes (light blue) compared to the other technologies. The proportion of detected sncRNAs in VASA-seq methods was higher than 10x Chromium and Smart-seq3, but lower than with Smart-seq-total (grey). **c**, Proportion of sncRNA biotypes captured for HEK293T cells across methods for reads mapped to all annotated genes. Only VASA-seq and Smart-seq-total detected a significant proportion of sncRNAs biotypes, with Smart-seq-total providing the best performance in terms of relative distribution of biotypes, followed by VASA-drop and VASA-plate. MiscRNA (brown), snoRNA (pink), Ribozyme(grey-green) and snRNA (red) took up the largest proportion of measured biotypes. **d**, The number of detected protein coding genes in HEK293T, for each method, is plotted against the number of reads (after quality filtering, adapter removal and homopolymer trimming), per cell across different downsampling thresholds. The saturation curves showed that VASA-seq was the most sensitive of the methods. Curvature of gene detection indicated that full complexity was not reached for the method when 75,000 reads were allocated to each cell. Only cells that were sequenced to at least 75,000 reads were used. **e**, Number of detected genes per cell for Smart-seq3 (red), Smart-seq-total (black) and VASA-plate (blue) when sequenced at a depth of approximately 750,000 reads per cell. Data in boxplot represent the 25%, median (centre) and 75% percentiles with minimum and maximum values. The number of cells sampled were *n* = 113 (Smart-seq3), 260 (Smart-seq-total) and 68 (VASA-plate). **f**, Percentage of sequenced reads with proper barcodes that survived trimming, rRNA filtering and mapping for each method using HEK2993T cells (VASA-plate, VASA-drop, 10x Chromium, Smart-seq3 and Smart-seq-total). **g**, Percentage of unspliced reads for each method for HEK293T cells. VASA-seq detected more unspliced reads (44.1–56.5%) than the alternative technologies (12.8–38.1%). Data in boxplot represent the 25%, median (centre) and 75% percentiles with minimum and maximum values. The number of cells sampled were *n* = 976 (10x Chromium), 117 (Smart-seq3), 260 (Smart-seq-total), 726 (VASA-drop) and 192 (VASA-plate).

To verify that the droplet compartments remained intact throughout consecutive steps of microfluidic processing with VASA-drop, we performed a species-mixing experiment with mouse embryonic stem cells (mESCs) and human HEK293T cells, which showed a heterotypic doublet rate of 3.08% (Fig. 1d and Extended Data Fig. 2a). We then compared the VASA-seq method to the widely used 10x Chromium^7^ droplet platform and the highly sensitive Smart-seq3 (ref. ^12^) and total RNA-seq Smart-seq-total^18^ plate-based workflows using HEK293T cells (Fig. 1e,f and Extended Data Fig. 2b–g). Both VASA-drop and VASA-plate exhibited homogeneous coverage across the body of protein-coding genes. In contrast, 10x Chromium had most of its reads located near the 3′ end. Smart-seq3 had a large bias toward the 5′ end for UMI-containing reads and toward the 3′ end for the remainder of the reads, which was also observed with Smart-seq-total (Fig. 1e).

Protein-coding genes were the most highly detected biotype across all methods. However, VASA-plate and VASA-drop both proportionally detected about twice as many long non-coding RNAs (lncRNAs) as 10x Chromium, Smart-seq3 and Smart-seq-total (Extended Data Fig. 2b). Only VASA-seq and Smart-seq-total detected short non-coding RNAs (sncRNAs) (1.4% for VASA-plate, 2.5% for VASA-drop and 6.7% for Smart-seq-total), mainly miscellaneous RNA (miscRNA), small nucleolar RNA (snoRNA), ribozymes and small nuclear RNA (snRNA) for VASA-seq and miscRNA and pre-transfer RNA (tRNA) for Smart-seq-total (Extended Data Fig. 2c).

Next, the HEK293T datasets for each method were downsampled to determine the gene detection sensitivity and saturation rates of each method for all annotated genes. VASA-drop showed the highest sensitivity, followed by VASA-plate, with 9,825 ± 280 and 9,480 ± 1,252 (mean ± s.d.) detected genes per cell, respectively, at a sequencing depth of 75,000 trimmed reads per cell. Both exhibited a higher gene detection rate than Smart-seq3 (9,022 ± 1,455 genes per cell) and 10x Chromium (8,342 ± 1,450 genes per cell) and outperformed Smart-seq-total (4,243 ± 512 genes per cell) (Fig. 1f). Similarly, both VASA-seq workflows showed superior detection of protein-coding genes (Extended Data Fig. 2d). For the highest read coverage in our sequenced dataset (750,000 trimmed reads per cell (Extended Data Fig. 2f), only for VASA-plate, Smart-seq3 and Smart-seq-total), VASA-plate and Smart-seq3 showed similar sensitivities (15,248 ± 1,092 and 14,631 ± 988 genes per cell, respectively), whereas Smart-seq-total showed lower sensitivity (7,403 ± 938 genes per cell) (Extended Data Fig. 2e).

Because VASA-seq detects full-length transcripts and larger amounts of unspliced RNA due to the poly(A) tailing of RNA fragments across the transcript length, it can detect nascent transcripts at higher rates than other methodologies. To quantify this, we assigned reads that aligned either to introns or to exon–intron junctions as unspliced, whereas reads that exclusively aligned to exons were considered as spliced. VASA-seq showed the highest proportion of unspliced reads at 44.1 ± 10.1% (VASA-plate) and 56.5 ± 3.1% (VASA-drop) compared to Smart-seq3 (14.8 ± 2.5%), 10x Chromium (17.7 ± 12.8%) and Smart-seq-total (38.1 ± 4.6%) (Extended Data Fig. 2g).

Overall, VASA-seq combines the throughput offered by the 10x Chromium droplet microfluidic platform, the high sensitivity of the Smart-seq3 methodology and the broad-spectrum capture of non-coding RNAs offered by Smart-seq-total in a single experimental workflow. In addition, the method preserves even coverage across the gene body for the unbiased capture of unspliced regions and splicing junctions.

### VASA-seq expands the list of cell-type-specific marker genes in the mouse embryo

**Fig. 2:**
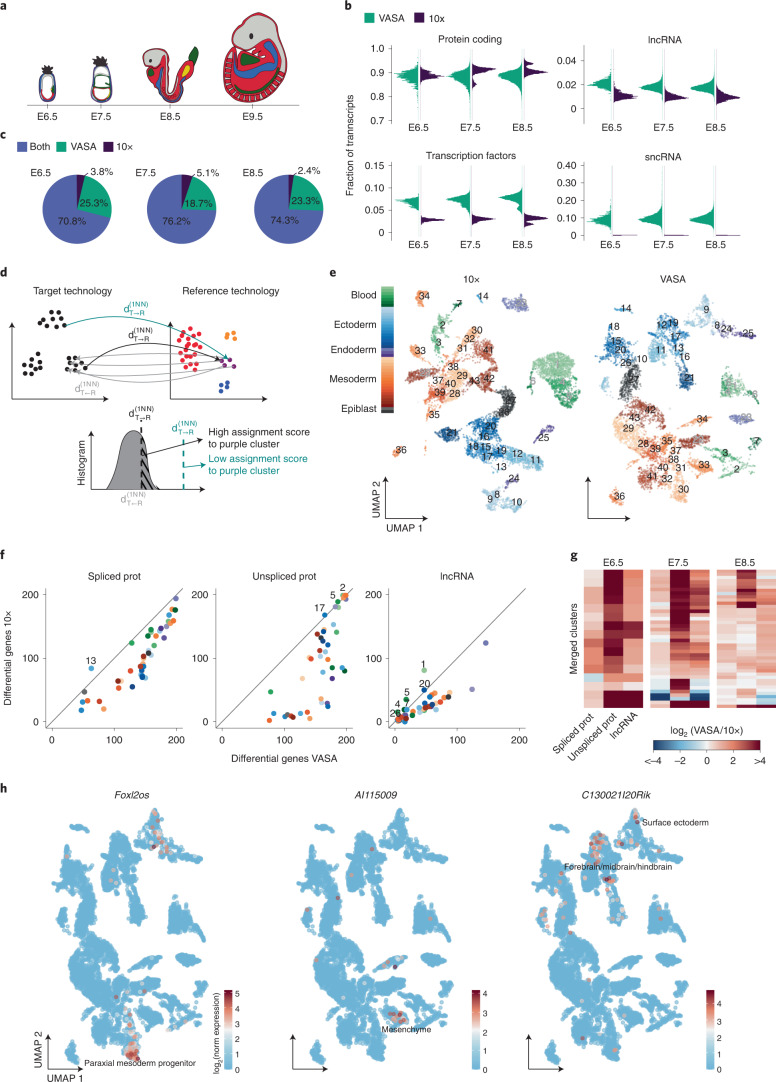
VASA-seq enables novel marker gene detection in the developing mouse embryo. **a**, Schematic figure of mouse embryo morphology at developmental stages E6.5, E7.5, E8.5 and E9.5 (left to right). **b**, Fraction of transcripts per biotype in VASA-seq compared to 10x Chromium for mouse embryos at each timepoint using the 20% terminal portion of genes. The comparison includes protein-coding genes (top-left panel), lncRNAs (top-right panel), TFs (bottom-left panel) and sncRNAs (bottom-right panel). **c**, Percentage of genes detected in VASA-seq compared to 10x for each timepoint using the 20% terminal portion of genes. 70.8–76.2% of the detected genes were shared between the methods; 18.7–25.3% were detected only in VASA-seq; and 2.4–5.1% were detected only in 10x. **d**, Strategy to transfer cluster identity from 10x Chromium or VASA-seq (reference technology) to VASA-seq or 10x Chromium (target technology) at the single-cell level. First, for a given cluster in the reference technology, a background histogram of the distances between cells in that cluster and their corresponding first nearest neighbor in the target technology is obtained (gray arrows and gray histogram). Next, each cell in the target technology is assigned to the cluster of its nearest neighbor cell in the reference technology (black and green arrows) with a score equal to the area under the left curve resulting from the intersection between the cell–cell distance and the corresponding background histogram (dashed area). This procedure is then repeated for all clusters in the reference technology. **e**, UMAP of E8.5 mouse embryo cells from 10x Chromium (*n* = 9,358) and VASA-seq (*n* = 7,899) that were part of equivalent clusters using the 20% terminal portion of genes. Clusters that are detected in both technologies are marked with numbers 1–43, and each cluster is colored according to the cell type category: green, blood; blue, ectoderm; purple, endoderm; orange, mesoderm; gray, epiblast. Gray fill in cluster label indicates extra-embryonic contribution; black fill indicates embryonic contribution. **f**, Scatter plot showing the number of differentially expressed genes per cluster in VASA-seq (*x* axis) versus 10x Chromium (*y* axis) for spliced protein-coding genes (left panel), unspliced protein-coding genes (middle panel) and lncRNAs (right panel) using the 20% terminal portion of genes. Numbers indicate clusters where a higher number of marker genes were detected in 10x Chromium. Clusters are colored according to the cell type category: green, blood; blue, ectoderm; purple, endoderm; orange, mesoderm, gray, epiblast. **g**, Heat maps showing the ratio of differential upregulated genes (log_2_ fold change >2 and *P* < 0.01), per cluster, between VASA-seq and 10x Chromium using the 20% terminal portion of genes. Columns display spliced protein-coding genes (left panel), unspliced protein-coding genes (middle panel) and lncRNAs (right panel), and rows are clusters. Red color indicates when marker genes are more predominantly detected in VASA-seq; blue color indicates when higher numbers of marker genes are detected in 10x Chromium. The statistical test used was a two-sided *t*-test, and *P* values were uncorrected for multiple comparisons. **h**, Examples of newly detected unspliced lncRNA marker genes in VASA-seq for E8.5: *Foxl2os* in paraxial mesoderm progenitors (left panel), *AI115009* in mesenchyme (middle panel) and *C130021I20Rik* in forebrain/midbrain/hindbrain and surface ectoderm (right panel).

**Extended Data Fig. 3:**
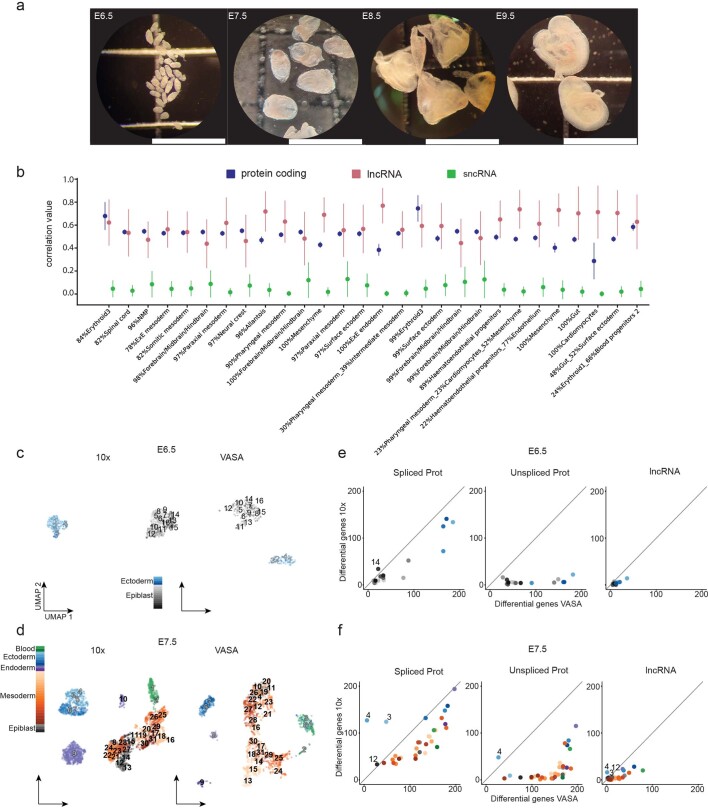
Mouse gastrulation and organogenesis atlas. **a**, Brightfield microscope images of the embryos collected before dissociation. Two collections were performed for E6.5 (39 embryos total), whereas single collections were performed for E7.5 (8 embryos total), E8.5 (7 embryos total) and E9.5 (6 embryos total). Scale indicates a 1 mm scale for the background gridlines. **b**, Average gene expression correlation values (*r*^2^) per biotype across equivalent clusters between 10x Chromiumand VASA-seq at stage E8.5. *n* number of cells were 8,365 (VASA-seq) and 9,939 (10x). The equivalent clusters are annotated by using the percentage of cells assigned to a cell type in 10x Chromium. Only cell types present in more than 30% of the equivalent cluster are indicated. The points are the mean and standard error of the mean obtained by bootstrapping genes for each equivalent cluster and biotype 1000 times are represented. **c**, UMAP of E6.5 mouse embryo cells from 10x (*n* = 640) and VASA-seq (*n* = 298) that were part of equivalent clusters. Clusters that are detected in both technologies are marked with numbers 1–16 and each cluster is colored according to the cell type category: blue = ectoderm and grey = epiblast. Grey fill in cluster label indicates extra-embryonic contribution, black fill indicates embryonic contribution. **d**, UMAP of E7.5 mouse embryo cells from 10x (*n* = 3,319) and VASA-seq (*n* = 1,892) that were part of equivalent clusters. Clusters that are detected in both technologies are marked with numbers 1–38 and each cluster is colored according to the cell type category: green = blood, blue = ectoderm, purple = endoderm, orange = mesoderm and grey = epiblast. Grey fill in cluster label indicates extra-embryonic contribution, black fill indicates embryonic contribution. **e**, Scatter plot showing the number of differentially expressed genes per cluster at E6.5 in VASA-seq (*x* axis) vs. 10x Chromium (*y* axis) for spliced protein coding (left panel), unspliced protein coding (middle panel) and lncRNA (right panel) counts. Numbers indicate clusters where a higher number of marker genes were detected in 10x. Clusters are colored according to the cell type category: blue = ectoderm and grey = epiblast. **f**, Scatter plot showing the number of differentially expressed genes per cluster at E7.5 in VASA-seq (*x* axis) vs. 10x Chromium (*y* axis) for spliced protein coding (left panel), unspliced protein coding (middle panel) and lncRNA (right panel). Numbers indicate clusters where a higher number of marker genes were detected in 10x. Clusters are colored according to the cell type category: green = blood, blue = ectoderm, purple = endoderm, orange = mesoderm and grey = epiblast.

Next, we used these advantages to extend and improve current atlases of mouse development. We used VASA-drop (referred to as VASA-seq in the remainder of the manuscript) to generate a single-cell total RNA-seq atlas of murine gastrulation and early organogenesis, with a total of 33,662 single cells sequenced from mouse embryonic post-implantation stages E6.5, E7.5, E8.5 and E9.5 (Fig. 2a and Extended Data Fig. 3a). The VASA-seq datasets from post-implantation E6.5, E7.5 and E8.5 were directly compared to a reference dataset generated using the 10x Chromium platform^24^.

Proportionally, VASA-seq detected a slightly lower fraction of protein-coding transcripts, but lncRNAs and transcription factors (TFs) were detected at about 2–3-fold-higher levels, whereas sncRNAs were captured only in the VASA-seq dataset (Fig. 2b). Overall, most genes were identified in both methods across timepoints (70.8–76.2%) (Fig. 2c), but 18.7–25.3% of the genes were detected only in the VASA-seq dataset, whereas a much smaller fraction was observed uniquely in the 10x Chromium dataset (2.4–5.1%).

To explore whether our total scRNA-seq atlas provided more marker genes for different cell types, we identified groups of equivalent cell clusters present in both VASA-seq and 10x Chromium and compared them through differential gene expression analysis, using only the reads that map to the 3′ terminal 20% of the gene bodies in both technologies (Fig. 2d,e and Extended Data Fig. 3b–d). For E8.5 embryos, we identified 43 equivalent clusters shared between the 10x Chromium and the VASA-seq datasets, allowing for systematic differential expression analysis for spliced/unspliced protein-coding transcripts as well as lncRNAs. Overall, VASA-seq detected a higher number of differentially upregulated genes (log_2_ fold change >2 and *P* *<* 0.01) for most equivalent comparisons with 10x Chromium (Fig. 2f,g and Extended Data Fig. 3e,f). Based on previous cell type annotations^24^, examples include the detection of *Foxl2os* as a paraxial mesoderm progenitor marker, *AI115009* as a marker for mesenchyme and *C130021I20Rik* as a specific marker for forebrain/midbrain/hindbrain and surface ectoderm (Fig. 2h). Comprehensive lists of all equivalent cluster markers are presented in Supplementary Table 2.

These results demonstrated that VASA-seq could expand the list of known marker genes, especially for unspliced protein-coding and lncRNA genes.

### Histone genes as in vivo markers for cycling cells

**Fig. 3:**
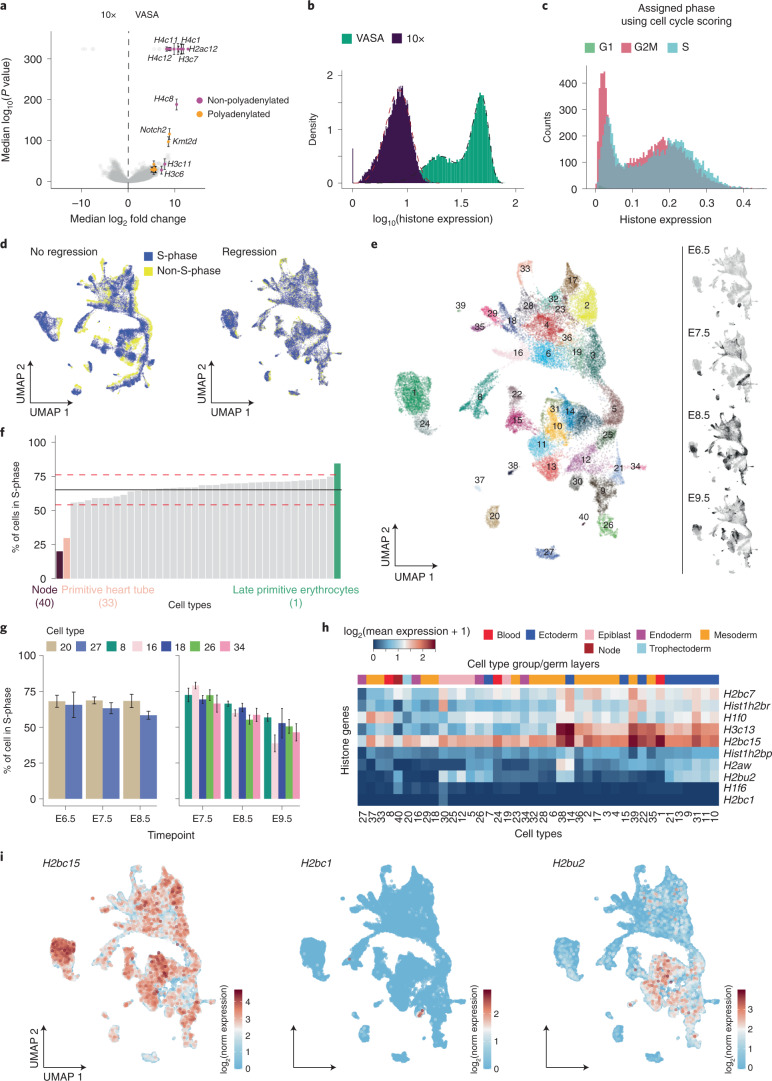
Histone gene expression robustly identifies cycling cells. **a**, Volcano plot showing differentially expressed genes between VASA-seq (right, positive values) and 10x Chromium (left, negative values). Genes that are always highly differentially expressed across timepoints and have a log_2_ fold change >4 and *P* < 0.001 are colored; purple color indicates non-polyadenylated, and orange color indicates polyadenylated genes. Many of the differentially expressed genes enriched in the VASA-seq dataset are histone genes. The statistical test used was a two-sided *t*-test, using uncorrected *P* values for multiple comparisons. **b**, Histogram showing the distribution of histone gene expression in VASA-seq compared to 10x Chromium. The overlayed dashed black line shows a bimodal Weibull distribution, and the dashed red line shows a single Weibull distribution. **c**, Histogram showing the distribution of histone gene expression in VASA-seq labeled with the estimated cell cycle phase using the expression of S and G2M genes for scoring. Detected histone expression in S-phase does not correlate with predictive cell cycle estimation. **d**, Cells are identified as cycling/S-phase (blue) and non-cycling (yellow) based on the total histone gene expression shown in Fig. 3b. UMAP of the VASA-seq embryonic atlas before (left panel) and after (right panel) removal of cell cycle genes. **e**, Cell type annotated UMAP of the aggregated VASA-seq dataset after removal of cell cycle genes. Each color and number represent a cell type, called manually based on marker gene expression for each Leiden cluster. Smaller panels (right) highlight cells sampled at each timepoint (E6.5, E7.5, E8.5 and E9.5) in black. In total, 40 different cell types were identified: 1-erythropoiesis (expansive, S-phase), 2-somites, 3-paraxial mesoderm, 4-intermediate mesoderm I, 5-caudal epiblast, 6-lateral plate mesoderm/intermediate mesoderm primordium, 7-spinal cord (differentiated neurons), 8-endothelium, 9-preplacodal/placodal region, 10-rhombomeres (hindbrain), 11-forebrain/hindbrain (isthmus), 12-epiblast (E7.5), 13-forebrain, 14-spinal cord (differentiated neurons), 15-neural crest, 16-allantois, 17-cranial mesoderm, 18-lateral plate mesoderm, 19-early caudal epiblast, 20-trophectoderm, 21-dorsal surface ectoderm, 22-anterior neural crest, 23-pharyngeal arches, 24-primitive erythroid progenitors, 25-caudal epiblast (E7.5), 26-endoderm, 27-visceral endoderm, 28-first heart field, 29-myofibroblasts, 30-epiblast (E6.5), 31-spinal cord (cycling progenitors), 32-pharyngeal arches, 33-primitive heart tube, 34-outflow tract, 35-secondary heart field, 36-intermediate mesoderm I, 37-parietal endoderm, 38-pro-nephros, 39-mesodermal unknown and 40-node. **f**, Percentage of cycling/S-phase cells per cell type. Average number of cycling cells is 65% (black line) ± 11% (red dashed lines) across all cell types. Late primitive erythrocytes (green) diverge from the average by having 84% of the cells in S-phase. Node cells (brown) and primitive heart tube (pink) have much fewer cells in S-phase—20% and 30%, respectively. **g**, Plots showing the percentage of cells in S-phase per cell type that spans over three timepoints (E6.5–E8.5, left panel; E7.5–E9.5, right panel). Trophectoderm (light brown) had an unchanged pattern, whereas endothelium (green), allantois (pink), lateral plate mesoderm (blue), endoderm (light green), visceral endoderm (light blue) and outflow tract (dark pink) all had a decreasing fraction of cycling cells as time passes. Allantois has the biggest difference, with 38% cycling in E9.5 compared to 79% in E7.5. The points are the mean and standard error of the mean obtained by bootstrapping the percentage of cells in S-phase for each equivalent cluster and biotype 1,000 times. The number of cells were: *n* = 140, 32 for cluster 20 and 27 at timepoint E6.5, respectively; *n* = 105, 340, 314, 392, 156, 171 and 69 for clusters 8, 16, 18, 20, 26, 27 and 34 at E7.5, respectively; *n* = 810, 552, 331, 117, 284, 339 and 121 for clusters 8, 16, 18, 20, 26, 27 and 34 at E8.5, respectively; and *n* = 345, 78, 30, 117 and 71 for clusters 8, 16, 18, 26 and 34 at E9.5, respectively. **h**, Heatmap showing differentially expressed single annotated histone genes. Rows display genes, and columns display cell types. Cell type categories/germ layers can be identified by color above the heat map. **i**, Example of marker histone gene expression plotted on the UMAP; red represents high expression, and blue represents low expression. *H2bc15* is highly expressed in most cell types but absent in certain cell types. *H2bc1* is solely expressed in the early epiblast (E6.5, cell type 30), whereas *H2bu2* is specific to the ectoderm germ layer and epiblasts (cell types 12 and 30).

To further identify global gene signatures intrinsic to VASA-seq, we performed differential gene expression analysis by comparing the mean expression values for all genes across equivalent clusters and timepoints. This analysis identified a subset of genes that were significantly higher expressed in VASA-seq (22 genes; log_2_ fold change >4 and *P* < 0.001), of which many were canonical histone genes (Fig. 3a and Supplementary Table 3). Consistently, most of the highly differentially expressed genes in the VASA-seq dataset are classified as non-polyadenylated^29^ (Fig. 3a).

**Extended Data Fig. 4:**
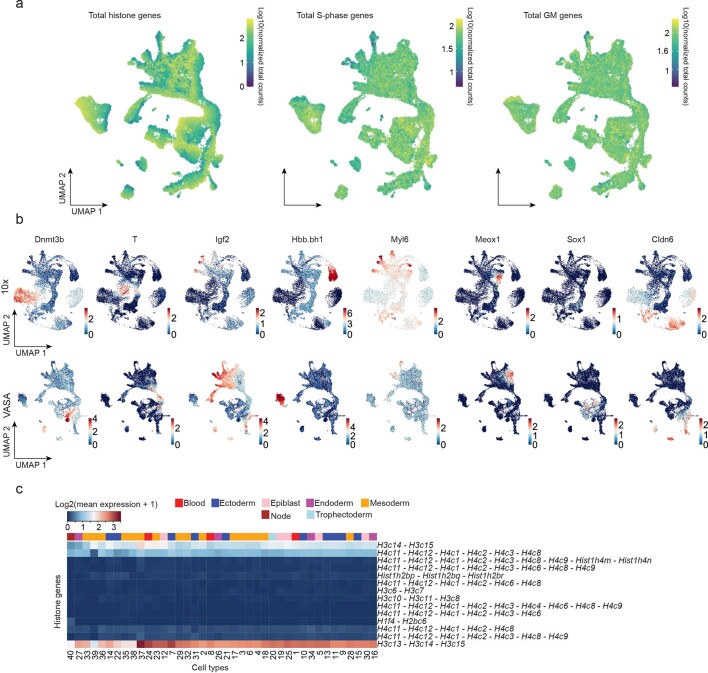
Histone expression and regression of cell-cycle. **a**, UMAPs showing the log10 total counts for histone genes (left panel), S-phase genes (middle panel) and GM genes (right panel). Only cell cycle scoring using solely histone genes shows a clear cell cycle segregation in VASA-seq. **b**, Core expression of cell-type specification markers during gastrulation and early organogenesis projected on the 10x Chromium and regressed VASA-seq UMAP. **c**, Heatmap showing differentially expressed multi annotated histone genes. Rows display genes, and columns display cell types. Cell type categories/germ layers are colored above the heatmap.

We reasoned that histone gene expression could be further used to identify cell cycle state, because most canonical histone genes are strongly upregulated during the S-phase^30^. A histogram of total histone gene expression per cell revealed a bimodal distribution for VASA-seq, in contrast to 10x Chromium (Fig. 3b). Detection of S-phase using canonical cell cycle gene expression^31^ did not overlap with histone content measurements, illustrating their benefit to confidently assign cell cycle phase in total RNA-seq datasets (Fig. 3c). We further embedded all cells from the different timepoints into a single UMAP^32^ and visualized the total expression of histone genes across the dataset (Extended Data Fig. 4a). Cells with high histone expression were clearly segregated in the uniform manifold approximation and projection (UMAP) from cells with low histone expression, a feature that was not detected using standard scRNA-seq cell cycle scoring methods (Extended Data Fig. 4a). The bimodal distribution of histone expression in the VASA-seq datasets enabled the classification of cells as being in S-phase (high total histone expression) or non-S-phase (low total histone expression) (Fig. 3d, left panel). Differential gene expression analysis between S-phase and non-S-phase cells was performed for either pooled or separate timepoints, which provided us with an extended list of cell cycle genes co-expressed with histones during mouse embryonic development (Supplementary Table 4).

We then regressed out cell cycle effects by removing the cell cycling genes from our dataset and produced an improved UMAP with reduced cell cycle patterning (Fig. 3d, right panel). We clustered the regressed data using the Leiden algorithm and assigned a cell type annotation to each cluster based on markers obtained through differential gene expression (Fig. 3e, Extended Data Fig. 4b and Supplementary Table 5). Next, we investigated if certain cell types were cycling more frequently. The proportion of cells in S-phase for each cell type in the mouse embryo was 65 ± 11%. However, some cell types displayed higher proportions of cells in S-phase, such as late primitive erythrocytes (84%), whereas node cells and cells from the primitive heart tube (PHT) showed lower proportions of cycling cells, with 20% and 30% of the cells in S-phase, respectively (Fig. 3f), consistent with results obtained using cell cycle reporter cell lines^33^. We also explored if the percentage of cells in S-phase changed for specific cell types across the probed developmental timepoints. We identified seven cell types that had at least 30 cells in each of three consecutive sampled timepoints: endothelium (cell type 8), allantois (cell type 16), lateral plate mesoderm (cell type 18), trophectoderm (cell type 20), endoderm (cell type 26), visceral endoderm (cell type 27) and outflow tract (cell type 34). In this subset, only the trophectoderm showed unaltered proportions of cells in S-phase from E6.5 to E8.5 (Fig. 3g, left panel). The other six cell types showed a reduction in the number of cells in S-phase across timepoints, with the allantois showing the most striking decrease from 79% to 38% between E7.5 and E9.5 (Fig. 3g, right panel).

Additionally, we performed differential histone gene expression analysis between cell types (Supplementary Table 6). Because histones from the same family (H1, H2a, H2b, H3 and H4) have extensive sequence similarity, not all reads could be uniquely assigned to a single histone gene. We found ten single-annotated (Fig. 3h) and 14 multi-annotated (Extended Data Fig. 4c) genes significantly upregulated in at least one cell type (log_2_ fold change >2; *P* < 0.01). Some histone genes showed germ layer and/or cell-type-specific expression. For example, *H2aw* was upregulated in the ectoderm. *H2bc15* was ubiquitously expressed in most cell types but absent in the node (cell type 40) and the visceral endoderm (cell type 27) (Fig. 3i, left panel). *H2bc1* expression was detected only in epiblast at E6.5 (cell type 30) (Fig. 3i, middle panel). *H2bu2* displayed specific gene expression in the ectoderm germ layer and epiblast (cell types 12 and 30) (Fig. 3i, right panel).

In conclusion, VASA-seq detected a high number of histone genes that enabled robust cell cycle and cell-type-specific histone usage determination across the dataset.

### Increased intron coverage with VASA-seq allows for improved RNA velocity estimates

**Fig. 4:**
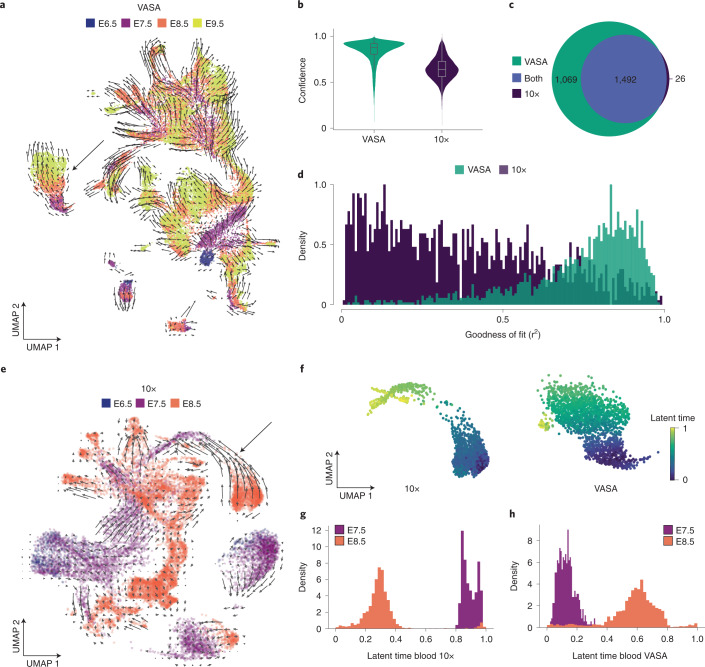
Increased intronic capture with VASA-seq improves RNA velocity measurements. **a**, UMAP of all four timepoints for VASA-seq (E6.5–E9.5). Velocity is shown as arrows and each timepoint as a separate color. The black arrow indicates blood maturation trajectory. **b**, Violin plot of confidence values for VASA-seq in green (left panel) and 10x Chromium in dark purple (right panel). Only the equivalent E6.5, E7.5 and E8.5 timepoints are included in the comparison. Average RNA velocity confidence was 0.84 ± 0.12 (s.d.) for VASA-seq and 0.65 ± 0.12 (s.d.) for 10x. *n* number of cells were 21,497 (VASA-seq) and 16,945 (10x Genomics Chromium). Data in the box plot represent the 25%, median (center) and 75% percentiles with minimum and maximum values. **c**, Venn diagram showing the significant genes, according to the scVelo package, for VASA-seq and 10x Chromium. In all, we found 1,492 genes that were significant in both datasets, 1,069 that were significant only in VASA-seq and 26 that were significant only in 10x. **d**, Histograms showing goodness of fit (r^2^) for the 1,492 genes that were significant in both VASA-seq and 10x Chromium. Average values were 0.74 ± 0.18 (s.d.) for VASA-seq and 0.38 ± 0.25 (s.d.) for 10x Chromium. **e**, UMAP of the equivalent 10x Chromium dataset (E6.5, E7.5 and E8.5) after filtering. Velocity is shown as arrows and each timepoint as a separate color. The black arrow indicates blood maturation trajectory. **f**, Predicted latent time projected on the blood and erythroid progenitor subsets, showing incorrect temporal prediction of blood maturation in the 10x dataset but not in the VASA-seq dataset. **g**, Histogram across latent time labeled with developmental timing of the embryos using the 10x Chromium dataset, showing incorrect temporal prediction of blood development using RNA velocity computation in dynamic mode. **h**, Histogram across latent time labeled with developmental time of the sampled embryos in the VASA-seq dataset, showing accurate prediction of blood development progression via RNA velocity computation in dynamic mode.

**Extended Data Fig. 5:**
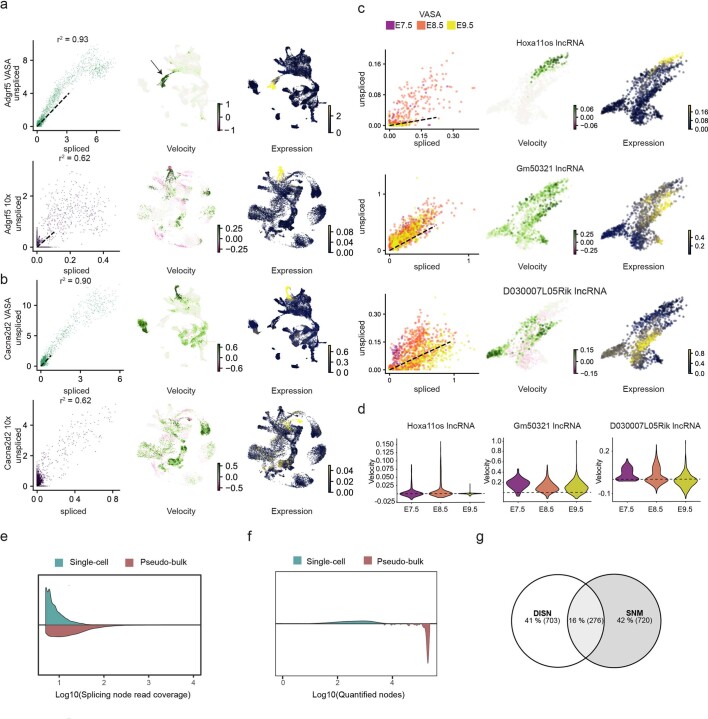
RNA velocity and global splicing marker analysis. **a**, Velocity of *Adgrf5* shown on diagrams of spliced vs. unspliced counts, along with UMAPs highlighting velocity and expression for the gene for VASA-seq (top) and 10x Chromium (bottom) showing both induction and repression in the endothelium for VASA-seq with high goodness of the fit. Goodness of the fit are values approximately one SD above average for each method to show genes that are good in both datasets. Black arrow indicates the endothelium in the VASA-seq dataset. **b**, Velocity of *Cacna2d2* shown on diagrams of spliced vs. unspliced counts, along with UMAPs highlighting velocity and expression for the gene for VASA-seq (top) and 10x Chromium (bottom) showing induction of the gene in the Primitive heart tube and first heart field for VASA-seq. Goodness of the fit are values approximately one SD above average for each method to show genes that are good in both datasets. **c**, Velocity of lncRNAs with unspliced molecules uniquely detected in the VASA-seq dataset for the endothelium. Phase diagrams of spliced vs. unspliced counts, along with UMAPs highlighting velocity and expression for VASA-seq show early induction of *Hoxa11os* in the the yolk sac, followed by induction of *Gm50321* across the endothelium (yolk sac and embryonic) and selective repression of *D030007L05Rik* at E9.5. Dots in the diagram are labelled according to developmental time points. **d**, Violin plot of the velocities across timepoints E6.5, E7.5 and E8.5 in the endothelium for the VASA-seq dataset showing differential induction and repression for lncRNAs. Dashed line indicates null velocity. **e**, Violin plot showing the distribution of coverage values obtained for splice nodes when computed at single-cell or pseudo-bulk level. **f**, Violin plot showing the number quantified spliced nodes (read coverage>5) obtained when quantified at the single-cell or pseudo-bulk level. **g**, Euler diagram showing the splicing node intersection between the DISN and SNM sets.

The large proportion of unspliced transcripts detected with VASA-seq suggested that RNA velocity profiles^26^, calculated using the ratio of unspliced-to-spliced counts for each gene, could be enhanced using this method. We, therefore, computed the velocities and confidence values using the scVelo package^25^ in stochastic mode for all cells across all four timepoints (E6.5–E9.5). The velocity vector directions clearly followed the consecutive timepoints and cell type progression in the UMAP, recapitulating previously characterized trajectories in the developing mouse embryo (Fig. 4a). To contrast with the equivalent 10x Chromium dataset, we repeated the analysis for both datasets using the E6.5, E7.5 and E8.5 timepoints. The RNA velocity vectors for VASA-seq had higher confidence metrics overall (0.84 ± 0.12) compared to 10x Chromium (0.65 ± 0.12) (Fig. 4b), highlighting higher average correlation of the velocity vectors for a given cell and its neighbors. Next, we extracted the number of genes that contributed significantly to the RNA velocity vectors. We found that most significant genes were shared between the methods (1,492). However, VASA-seq detected a large number of additional genes (1,069) that contributed to the RNA velocity vector (Fig. 4c and Supplementary Table 7). For the genes that were shared between both methods, we quantified the goodness of fit (r^2^) to the gene phase diagrams with the prediction made by scVelo (Fig. 4d). The stochastic model of the scVelo package fitted better to VASA-seq in terms of goodness of fit (0.74 ± 0.18) compared to the 10x Chromium data (0.38 ± 0.25). Examples of genes with an r^2^ about 1 s.d. above average for both VASA-seq and 10x Chromium are shown in Extended Data Fig. 5a. To determine whether these measurements would enable a more accurate trajectory prediction across our atlas, the velocity vectors from the 10x Chromium dataset were projected on the UMAP spanning the developmental timepoints E6.5, E7.5 and E8.5. This analysis revealed a discrepant trajectory across blood maturation (Fig. 4e) that was not observed in our dataset (Fig. 4a). Latent time predictions using scVelo’s dynamical modeling on the blood cell types across E7.5 and E8.5 further highlighted trajectory inconsistencies for the 10x Chromium dataset (Fig. 4f,g), which has previously been associated with confounding effects from multiple rate kinetics genes in the overlapping first and second blood waves^34^. These observations were not replicated with VASA-seq, which accurately reported on blood maturation across physically sampled timepoints (Fig. 4h). These findings highlight the benefits of more sensitive RNA velocity measurements using VASA-seq to agnostically identify trajectories across cell types. Based on the capture of non-coding species across their gene body using VASA-seq, lncRNA kinetics across tissues can be determined. For example, the endothelium showed (1) the induction of *Hoxa11os* in the yolk sac at E7.5 and E8.5; (2) the induction of *Gm50321* at E7.5 and split induction and repression at E8.5 and E9.5; and (3) the induction of *D030007L05Rik* at E7.5 and progressive repression across E8.5 and E9.5 (Extended Data Fig. 5c,d). These observations could not be replicated in the 10x Chromium dataset because unspliced molecules for these lncRNAs could not be detected.

Therefore, VASA-seq showed better reconstruction of RNA velocity vectors guiding differentiation trajectories and identification of novel gene expression dynamics.

### Comprehensive profiling of AS across mouse gastrulation and early organogenesis

**Fig. 5:**
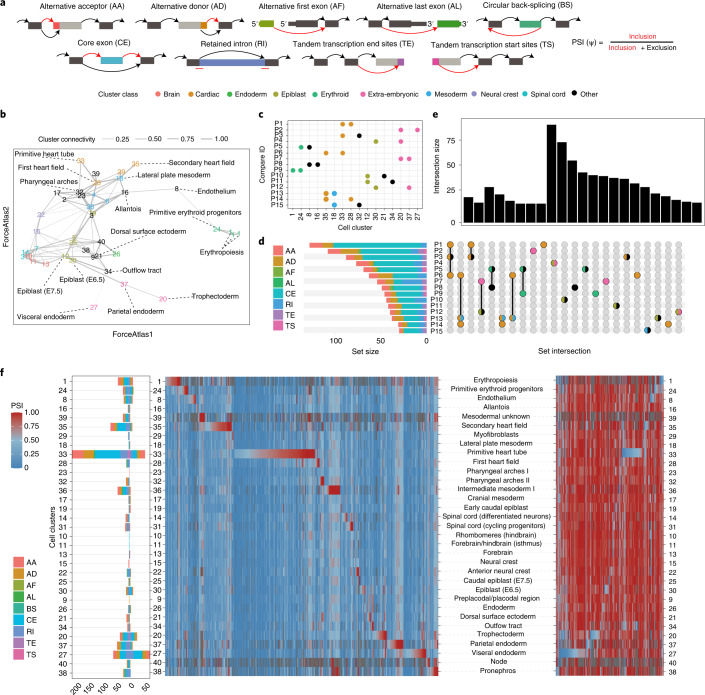
Landscape of AS events across cell types involved in gastrulation and early organogenesis. **a**, Schematic representation of the different types of AS nodes categorized using Whippet. Colors highlight the exonic parts that correspond to the indicated splicing nodes. Arrows indicate different possible splice junctions that can be connected to the nodes. Red arrows represent inclusion events; black arrows represent exclusion events. **b**, PAGA projecting cell types onto a force field revealing connectivity between cell clusters. Edge width represents the connectivity values between cell clusters. Selected cell types are highlighted by color. **c**, Pairwise comparisons that exhibited the highest number of DISNs are represented by a dot plot, where the position of each point on the *x* axis indicates the cell clusters that were involved in each comparison (P1–P15) across the *y* axis. Color codes correspond to clusters in **b**. **d**, Number of DISNs found for each pairwise comparison (P1–P15). Color codes of the stacked bar plot indicate the abundance of different classes of splicing nodes that were found to be differentially included across the comparisons. **e**, Upset plot showing set interactions across the group of DISNs found for each pairwise comparison. Bar plot indicates the size of each intersection, and their composition is described by the bottom panel. **f**, Splice node makers identified for each cell cluster. The left panel shows the number of SNMs found for each cell cluster. The direction of the stacked bars (left or right) indicates if these markers were found with positive or negative Z-score values, respectively. The right panel corresponds to heat maps displaying ψ values per cell type (blue to red scale) for different SNMs. Heat map on the left represents inclusion SNMs, and the heat map on the right represents exclusion SNMs.

The ability to profile full-length transcripts at scale using VASA-seq allows for the identification of AS patterns across cell types by quantifying the inclusion rates of non-overlapping exonic parts, herein referred to as ‘splicing nodes’. Every splicing node is associated with different types of AS, alternative transcriptional start sites or alternative polyadenylation events, and their inclusion rates are calculated as percent-spliced-in (ψ) values, which is quantified by taking the ratio of reads that support the inclusion of a given splicing node (Fig. 5a). To quantify AS patterns, we used Whippet, a computationally lightweight and accurate quantification method, previously integrated in computational workflows dedicated to process scRNA-seq data^35,36^. Because splicing node coverage is limited at the single-cell level (Extended Data Fig. 5d), we implemented a pseudo-bulk pooling approach, developed as part of MicroExonator^35^, where reads from the same cell type are pooled in silico before differential splicing node quantification. Pooling reads into pseudo-bulks from each cell type substantially increased our ability to quantify splicing nodes (Extended Data Fig. 5e–g). To detect differentially included splicing nodes (DISNs) across cell types, we implemented a method developed as part of MicroExonator to detect robust AS changes across pairwise comparisons of closely related cell types. For this purpose, we computed the *k*-nearest neighbor connectivity values across cell types to generate a coarse-grained graph with partition-based graph abstraction (PAGA)^37^(Fig. 5b). This enabled us to compute 72 pairwise comparisons between related cell types, from which we identified a total of 979 DISNs (Supplementary Table 8). We found that 45.8% of DISNs were core exon (CE) nodes, which correspond to cassette exons involved in exon skipping, the most abundant type of AS event across vertebrates^38^.

To further investigate the AS profile across cell types, we focused our analyses on the 15 pairwise comparisons that detected the highest amount of DISNs, which accounted for 67.6% of the total. These comparisons were overrepresented with cell types involved in heart morphogenesis, early gastrulation, extra-embryonic tissues and blood development, showing widespread involvement of AS in these processes. Again, CE was the most abundant type across DISNs, except for comparison 7 (trophectoderm (cell type 20) versus parietal endoderm (cell type 37)), where 40.7% of the DISNs were classified as retained intron (RI) (Fig. 5c,d). Further intersection analyses between the set of DISNs revealed differential splicing nodes that were recurrently detected across different comparisons. The biggest set of common DISNs was found across comparisons that share cell clusters, such as P1/P3/P6 or P6/P13/P14, which all correspond to cell types involved in heart development (Fig. 5e). However, 68.7% of the identified DISNs were found exclusively across individual pairwise comparisons, suggesting a prevalence of AS events that are specific to certain differentiation transitions.

To gain further insights of global splicing patterns in relation to cell types, we identified splicing nodes with ψ values that strongly deviated from the rest of the cell types and denoted these as splice node markers (SNMs) (Fig. 5f). In total, we identified 996 SNMs (Supplementary Table 9), 27.7% of which were also detected as DISNs (Extended Data Fig. 5g). In agreement with our previous analyses, we detected an elevated number of SNMs for cell types involved in heart development and early gastrulation. Among all the cell types, the PHT (cell type 33) had the most divergent splicing patterns and featured the highest number of SNMs (263), both included and excluded (Fig. 5f). Moreover, we found 132 SNMs for the second heart field (cell type 35), supporting the observation of extensive AS activity during heart morphogenesis. Extra-embryonic cell types that were sampled in the earlier timepoints mainly (E6.5 and E7.5), such as the trophectoderm (cell type 20), parietal endoderm (cell type 37) and visceral endoderm (cell type 27), also exhibited a higher-than-average proportion of SNMs (62, 56 and 132, respectively).

Taken together, we show that sequencing transcripts across their length with high cellular coverage using VASA-seq enabled the identification of extensive AS patterns during mouse development.

### AS analysis of blood and heart-related cell types

**Fig. 6:**
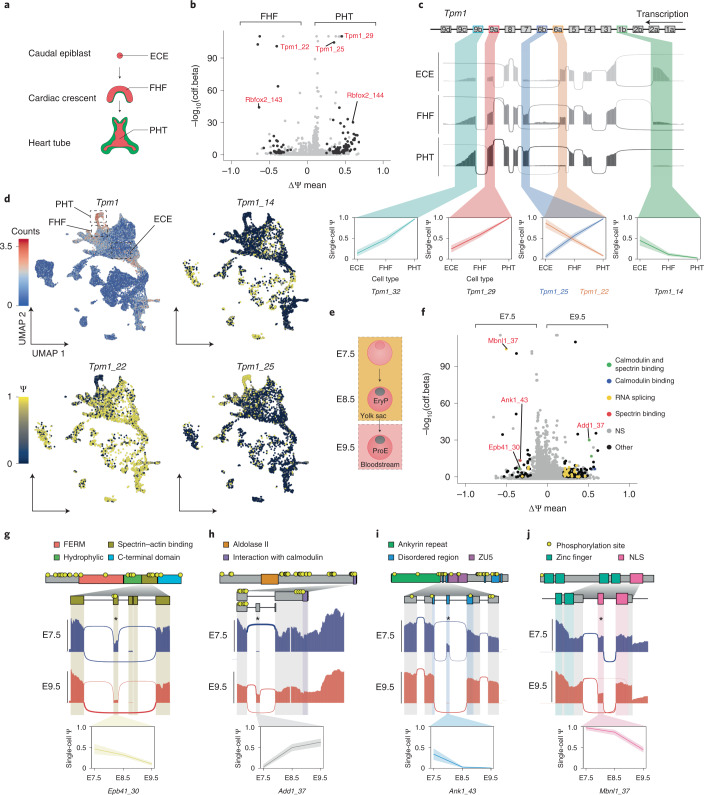
AS patterns across heart morphogenesis and blood formation. **a**, Schematic of mouse heart development. **b**, Volcano plot illustrating the DISNs detected between the PHT (positive ΔΨ values) and the FHF (negative ΔΨ values). **c**, Sashimi plot of *Tpm1* showing a coordinated AS switch from smooth muscle to striated muscle conformation after heart development from ECE to PHT. Box annotation on top illustrates exon order for *Tpm1*. Color-coding indicates the splicing node. Line in the bottom single-cell Ψ plots across cell types delineates the mean Ψ value, and the shading indicates the 95% confidence interval (CI). **d**, Single-cell gene expression UMAP plot for *Tpm1* (top left) and single-cell Ψ projection for *Tpm1_14, Tpm1_22* and *Tpm1_25* across the global splicing analysis. Each node is color-coded and highlighted in a single-cell line plot representing single-cell ψ values across all three clusters. **e**, Schematic representation of murine blood development throughout the profiled timepoints. **f**, Volcano plot illustrating the DISNs detected in the pairwise comparison between erythrocytes at E7.5 and E9.5. Color-coding indicates proteins with calmodulin-binding and/or spectrin-binding domains or RNA splicing proteins as determined by GO analysis. NS annotation stands for non-significant (gray color). **g**, Sashimi, domain annotation and line plots representing the skipping of exon 16 (*Epb41_30)* between E7.5 and E9.5. Line in the bottom single-cell Ψ plots across timepoints delineates the mean Ψ value at each timepoint, and the shading indicates the 95% CI. **h**, Sashimi, domain annotation and line plots representing the inclusion of *Add1_37* leading to a premature stop codon inclusion at E9.5 removing the C-terminus calmodulin-binding domain. Line in the bottom single-cell Ψ plots across timepoints delineates the mean Ψ value at each timepoint, and the shading indicates the 95% CI. **i**, Sashimi, domain annotation and line plots representing the gradual exclusion of the *Ank1_43* microexon in a disordered domain. Line in the bottom single-cell Ψ plots across timepoints delineates the mean Ψ value at each timepoint, and the shading indicates the 95% CI. **j**, Sashimi, domain annotation and line plots representing the gradual exclusion of the *Mbnl1_37* nuclear localization signal mediating the protein’s intracellular localization across timepoints. Line in the bottom single-cell Ψ plots across timepoints delineates the mean Ψ value at each timepoint, and the shading indicates the 95% CI.

Across all cell types, the PHT showed considerable AS signatures compared to the first heart field (FHF) (comparison 1; Fig. 5c–f). These changes occur while the heart undergoes extensive morphogenesis with the formation of the cardiac crescent, consisting of the FHF and second heart field (SHF) at E7.5, which subsequently re-arranges to form the PHT at E8.0 (ref. ^39^) (Fig. 6a).

**Extended Data Fig. 6:**
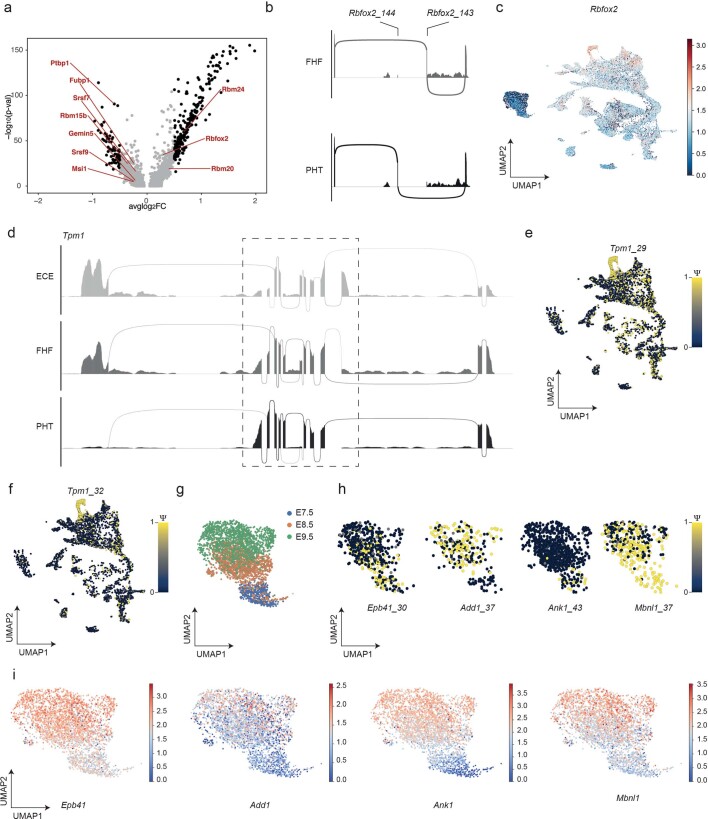
Heart and blood development reveal tissue-specific AS patterns across developmental trajectories. **a**, Differential gene expression analysis using a two-sided Wilcoxon rank sum test between the FHF (negative average log2 fold-change values) and the PHT (positive average log2 fold-change values) with differentially expressed RBPs highlighted. Significance levels are indicated by color (grey non-significant and black significant), and determined by the following threshold: |average log2 fold-change | > 0.5 and Bonferroni adjusted p-value < 1E-05). **b**, *Rbfox2*_143 and 144 mutually exclusive exon usage in the FHF and PHT respectively. **c**, *Rbfox2* gene expression across the atlas, log2 normalized values. **d**, *Tpm1* sashimi plot between the ECE, FHF and PHT, dashed square highlights the region of interest plotted in Fig. 6c. **e**, *Tpm1_29* single-cell PSI UMAP plot across the atlas highlighting a PHT specific core exon usage at the C-terminus. **f**, *Tpm1_32* single-cell PSI UMAP plot across the atlas highlighting a PHT specific core exon usage at the C-terminus. **g**, UMAP plot across timepoints depicting erythropoietic cell types. **h**, Single-cell Ψ UMAPs of *Epb41_30*, *Add1_37*, *Ank1_43* and *Mbnl1_37* depicting alternative exonic usage across blood maturation trajectories. **i**, Single-cell gene expression UMAP plot depicting differences in gene expression for *Epb41*, *Add1*, *Ank1* and *Mbnl1* illustrating differences in gene expression that differ from the AS patterns observed across blood maturation.

The detected splicing events for comparison 1 (Supplementary Table 10) were coordinated with the differential expression of heart-specific RNA-binding proteins (RBPs), such as *Ptbp1* (Extended Data Fig. 6a), that are likely orchestrating the observed AS events^40,41^. In addition to changes in gene expression for RBPs, a pair of mutually exclusive exons for *Rbfox2* (*Rbfox2_143/144*, commonly referred to as B40 and M43 in the literature) were among the most significant DISNs identified in the FHF to PHT comparison (Fig. 6b and Extended Data Fig. 6b,c). Our results showed that B40 and M43 were preferentially included in FHF and PHT cells, respectively, which is in line with previous findings^42^. In addition, tropomyosin 1 (*Tpm1*) stood out with three DISNs, a CE and a pair of mutually exclusive exons, which had some of the highest confidence levels detected (*Tpm1*_29, *Tpm1*_22 and *Tpm1*_25 corresponding to exons 9a, 6a and 6b, respectively). These splicing events are part of a coordinated transition between a smooth muscle and striated muscle program, orchestrated by PTBP1 and RBFOX2 (ref. ^43^). This transition was captured along a differentiation trajectory encompassing the early caudal epiblast (ECE), the FHF and PHT (Fig. 6c and Extended Data Fig. 5d), which also highlighted a switch for the N- (*Tpm1*_14, exon 1b) and C- (*Tpm1*_32, exon 9b) termini that modulate the protein’s interaction with actin and troponin^43,44^. Because *Tpm1* has many cell-type-specific isoforms^45^, we further visualized single-cell ψ values for the aforementioned splicing nodes on the UMAP, which showed cell-type-specific patterning across the atlas (Fig. 6d and Extended Data Fig. 6e,f).

At E7.25, primitive erythroids emerge from the blood islands in the yolk sac and enter the bloodstream at E9.0 (ref. ^46^) (Fig. 6e). The erythroid cytoskeleton then undergoes gradual rearrangements that increase their deformability when circulating in the narrow network of fetal vasculature, a change catalyzed by the adoption of the erythrocyte-specific transmembrane spectrin–actin backbone^47^. To determine if we could identify DISNs that mediate such rearrangements, we performed pairwise differential splicing analysis between erythroid cells from E7.5 (early progenitors, primitive erythroids) and E9.5 (early differentiating proerythroblasts, ProE) (Extended Data Fig. 6g). The analysis uncovered 210 DISNs that showed an enrichment for Gene Ontology (GO) terms relating to spectrin (GO:0030507; false discovery rate (FDR) = 4.8 × 10^−3^) and calmodulin (GO:0005516; FDR = 4.8 × 10^−3^) binding, suggesting extensive transmembrane cytoskeletal protein rearrangements (Fig. 6f and Supplementary Table 11). *Epb41*, a core member of the erythrocyte cytoskeleton^48^, showed a gradual exclusion of exon 16 (*Epb41*_30) across timepoints (Fig. 6g and Extended Data Fig. 6h,i). This domain contains two phosphorylation sites, directly interacts with spectrin and actin and has been shown to be gradually included at later timepoints, suggesting a narrow exclusion window for exon 16 in primitive erythroids as they enter the bloodstream. *Add1*, which binds to ɑ- and β-spectrin and caps actin to support the membrane-bound cytoskeleton, displayed the inclusion of a premature stop codon at E9.5 (*Add1*_37), hereby excluding a C-terminal calmodulin-binding domain that otherwise destabilizes its interaction with spectrin and F-actin upon calcium stimulation^49^ (Fig. 6h and Extended Data Fig. 6h,i). *Ank1*, which links the membrane to the underlying spectrin–actin filaments, had a skipped microexon (*Ank1*_43) at E9.5 that directly affected one of its intrinsically disordered regions (Fig. 6i and Extended Data Fig. 6h,i), which predominantly contain post-translational modification and protein–protein interaction sites^50^. The identified cytoskeletal splicing rearrangements were accompanied by the detection of AS motifs in RBPs known to be involved in terminal erythropoiesis (Fig. 6g; RNA splicing GO:0008380; FDR = 7.05 × 10^−6^). For example, *Mbnl1* (ref. ^51^), showed a skipped exon (*Mbnl1*_37) encoding for a nuclear localization signal (Fig. 6j and Extended Data Fig. 6h,i). Nuclear localization signal skipping of this exon leads to its localization in the nucleus and cytoplasm rather than exclusively in the nucleus^52^, likely affecting the spectrum of AS events depicted across early erythroid progenitor differentiation.

These results show that VASA-seq can inform on cell-type-specific gene function by measuring AS across cell types.

## Discussion

VASA-seq is a novel technology that enables the sequencing of the total transcriptome from single cells. The protocol demonstrates best-in-class RNA capture efficiency and provides full-length coverage of coding sequences and enriches for non-coding RNA biotypes. In our datasets, the latter were detected at much higher levels compared to current state-of-the-art methods: 10x Chromium^7^ and Smart-seq3 (ref. ^12^). VASA-seq also outperformed Smart-seq-total^18^ in terms of scalability, sensitivity, balance in gene body coverage and lncRNA detection. However, Smart-seq-total may complement our approach for the study of specific sncRNAs. VASA-seq does not rely on random priming, which has been shown to induce sequence-specific biases in transcriptome composition^53^. Fragment quantification is also ameliorated, as it employs UMI/UFI tagging across the whole gene body, and the reads retains strand specificity, which improves the quantification of overlapping transcripts^54^.

The excellent performance of the method was maintained for both plate-based (VASA-plate) and droplet-based (VASA-drop) formats in our benchmarking effort. However, a discrepancy arose for sncRNAs and unspliced molecules, which were detected at lower levels in VASA-plate compared to VASA-drop, possibly due to inefficient nuclei lysis in the plate experiment or different length exclusion during the DNA purification steps. On the other hand, rRNAs were not depleted as efficiently in the VASA-drop datasets, maybe because the increased barcode length for the method decreases the ability to exclude short ribosomal fragments that remain after depletion using DNA purification methods. Nevertheless, rRNA depletion in both VASA-seq methods outperformed Smart-seq-total. For integration with datasets generated with 3′ capture methods, we recommend using the 20% terminal fragments of gene bodies to generate a shared embedding.

The throughput of the method is an order of magnitude larger than previously described total RNA-seq methodologies^16–18^. This allowed us to generate a large-scale total-RNA-seq atlas to profile mouse gastrulation and early organogenesis. The high sensitivity and increased coverage of non-coding RNA molecules enabled us to expand the current list of cell-type-specific markers that will complement previous findings^20–24^. We further provide a detailed map of cell-type-specific AS events encompassing mouse development from E6.5 to E9.5, which underlined the predominance of alternative cassette exon usage throughout the timepoints investigated. Our resource provides a comprehensive analysis of AS during post-implantation mammalian development.

Furthermore, VASA-seq enables the accurate estimation of cell cycle stage from direct measurements of histone content. Because most histone genes are non-polyadenylated^55^ and because canonical histone expression is a marker for S-phase^30^, VASA-seq outperformed previous cell cycle scoring methods based solely on polyadenylated marker expression in our dataset. This is especially useful to determine cell cycling across developmental phases or between different populations of cells. The workflow also enables effective removal of cell cycle effects on profiled transcriptomes, which is important for cell type classification and unbiased analysis^56^.

Because VASA-seq captures RNA molecules across their entire length, RNA velocity predictions were ameliorated. This offers a resource for further explorations that go beyond transcriptional kinetics, such as the detection of splicing dynamics across developmental trajectories.

The modularity afforded by the microfluidic workflow will expand the number of single-cell assays that can be performed at high throughput. Indeed, consecutive injections of reaction mixes in droplets enables multi-step processes will benefit complex multi-omic workflows. Moreover, lower reagent costs due to smaller volumes, associated with droplet miniaturization^57^, and the lack of reliance on commercial kits for the VASA-drop workflow will enable inexpensive, large-scale, in-depth transcriptomic profiling at a cost of approximately $0.11 USD per cell for sequencing-ready libraries compared to $0.5 USD per cell for the 10x Chromium v2 kit^58^. The VASA-plate method has a library preparation cost of $0.98 USD, which is similar to the estimated range between $0.57 USD and $1.14 USD per cell for Smart-seq3 (ref. ^12^) (Supplementary Table 13).

## Methods

### Ethics statement

Experiments were performed in accordance with European Union guidelines for the care and use of laboratory animals and under the authority of appropriate United Kingdom governmental legislation. Use of animals in this project was approved by the Animal Welfare and Ethical Review Body for the University of Cambridge, and relevant Home Office license PPL (7677788) is in place.

### Cell lines

HEK293T cells were passaged every second day and cultured in T75 flasks. The culture media was DMEM-F12 (Thermo Fisher Scientific) supplemented with 10% heat-inactivated FBS (Thermo Fisher Scientific) and 100 U ml^−1^ of penicillin–streptomycin (Thermo Fisher Scientific). For passaging, the cells were washed with 10 ml of ice-cold 1× PBS (Lonza) twice. Then, 9 ml of PBS was added to the flask, and cells were detached by adding 1 ml of 10× trypsin-EDTA (Sigma-Aldrich) and incubated at 37 °C for 5 minutes. Trypsin-EDTA was then inactivated with 15 ml of DMEM-F12 + 10% FBS and incubated at 37 °C for 5 minutes. The cells were then pelleted at 300*g* for 3 minutes, and the supernatant was aspirated. After aspiration of the supernatant, the cells were washed twice in PBS and viability-assessed and counted before encapsulation.

mESCs were passaged every other day and cultured in 2i+LIF medium. In brief, DMEM/F-12 nutrient mixture without L‑glutamine (Thermo Fisher Scientific) and neurobasal medium without L‑glutamine (Thermo Fisher Scientific) in a 1:1 ratio, 0.1% sodium bicarbonate (Thermo Fisher Scientific), 0.11% bovine albumin fraction V solution (Thermo Fisher Scientific), 0.5× B-27 supplement (Thermo Fisher Scientific), 1× N-2 supplement (Cambridge Stem Cell Institute, made in-house), 50 µM 2-mercaptoethanol (Thermo Fisher Scientific), 2 mM L-glutamine (Thermo Fisher Scientific), 100 U ml^−1^ of penicillin–streptomycin (Thermo Fisher Scientific), 12.5 µg ml^−1^ of insulin zinc (Thermo Fisher Scientific), 0.2 µg ml^−1^ of mLIF (Cambridge Stem Cell Institute), 3 µM CHIR99021 (Cambridge Stem Cell Institute) and 1 µM PD0325901 (Cambridge Stem Cell Institute). Culture dishes were coated with 0.1% gelatine in PBS for at least 30 minutes. Cells were detached with 500 μl per six-well of Accutase (Merck) for 3 minutes at 37 °C. The detached cells were transferred into 9.5 ml of washing medium (DMEM/F-12 with 1% bovine albumin fraction V solution) and centrifuged at 300*g* for 3 minutes. The supernatant was aspirated, and the cell pellet was resuspended in 2i+LIF medium and re-plated at 80,000 cells per six-well. For the encapsulation process, the cells were washed twice in PBS, viability-assessed and counted before dilution to the correct concentration.

### Murine embryo collection and dissociation

Pregnant C57BL/6 female mice were purchased from Charles River Laboratories or obtained from natural mating of C57BL/6 mice in-house. Mice were maintained on a lighting regimen of 12-hour light/dark cycle with food and water supplied ad libitum. Detection of a copulation plug after natural mating indicated E0.5. After euthanasia of the females using cervical dislocation, the uteri were collected into PBS (Lonza) with 2% heat-inactivated FBS (Gibco, Thermo Fisher Scientific), and the embryos were immediately dissected and processed for scRNA-seq. Mouse embryos were dissected at timepoints E6.5, E7.5, E8.5 and E9.5, as previously reported^54^. Embryos from the same stage were pooled in a LoBind tube (Eppendorf). E8.5 and E9.5 embryos were cut into pieces under stereomicroscopy before collecting into a tube. The pooled samples were centrifuged at 300*g* for 5 minutes. The supernatant was aspirated, and 100–200 µl of TrypLE Express (Gibco) dissociation reagent was added to the samples. The tube was incubated at 37 °C for a minimum of 7 minutes (or until completely dissociated) in an orbital shaker. Subsequently, 1 ml of FBS was added to the tube to inactivate TrypLE. The sample was repeatedly centrifuged and washed with PBS before finally being resuspended in PBS supplemented with 0.04% BSA and filtered through a 40-µm Flowmi Tip Strainer (Thermo Fisher Scientific).

### VASA-plate: cell sorting in 384-well plates

Single cells were sorted into 384-well hardshell plates (BioRad) using a BD FACSJazz. Each well was pre-filled with 5 µl of mineral oil (Sigma-Aldrich) and 50 nl of CEL-seq2/SORT-seq^1,27^ primer with a concentration of 0.25 µM. Plates were sealed (Greiner, SILVERseal sealer, 676090) and spun down at 2,000 revolution centrifugal force (r.c.f). for 1 minute (Eppendorf 5810R) before being stored at −80 °C.

### VASA-plate: cell lysis and RNA fragmentation

All dispensions were carried out with a NanoDrop II (Innovadyne Technologies), all incubations with a GeneAmp PCR System 9700 Thermal Cycler (Applied Biosystems) and all spinning steps with an Eppendorf 5810R, unless otherwise specified. Next, 50 nl of lysis and fragmentation mix (3.4× First-Strand Buffer (Invitrogen), 1.2 mU of Thermolabile Proteinase K (New England Biolabs (NEB))) and 0.2% IGEPAL CA-630 (Sigma-Aldrich) were added to each well. Plates were sealed and spun down at 2,000 r.c.f. for 2 minutes. Lysis was carried out at 25 °C for 1 hour, followed by 55 °C at 10 minutes. Plates were snap-chilled on ice before fragmentation was carried out at 85 °C for 3 minutes. Plates were snap-chilled, spun down at 2,000 r.c.f. for 1 minute and stored on ice before next dispensation.

### VASA-plate: RNA repair and poly(A) tailing

Next, 50 nl of RNA repair and poly(A)-tailing mix (0.6× First-Strand Buffer (Invitrogen), 20 mM DTT (Invitrogen), 7.5 nM ATP (NEB), 37.5 mU of *E. coli* Poly(A) Polymerase (NEB), 50 mU of T4 PNK (NEB) and 10 mM RNaseOUT (Invitrogen)) were added to each well. Plates were sealed and spun down at 2,000 r.c.f. for 2 minutes. Repair and tailing were carried out at 37 °C for 1 hour. Plates were snap-chilled, spun down at 2,000 r.c.f. for 1 minute and stored on ice before next dispensation.

### VASA-plate: reverse transcription

Next, 50 nl of reverse transcription mix (2 mM (each) dNTP mix (Promega) and 0.8 U of SuperScript III (Invitrogen)) was added to each well. Plates were sealed and spun down at 2,000 r.c.f. for 2 minutes. Reverse transcription was carried out at 50 °C for 1 hour. Plates were snap-chilled, spun down at 2,000 r.c.f. for 1 minute and stored on ice before next dispensation.

### VASA-plate: second-strand synthesis

Next, 1,100 nl of second-strand synthesis mix (1.14× Second-Strand Buffer (Invitrogen), 0.23 mM (each) dNTP mix (Promega), 0.35 U of *E. coli* DNA Polymerase I (Invitrogen) and 20 mU of RNaseH (Invitrogen)) was added to each well. Plates were sealed and spun down at 2,000 r.c.f. for 2 minutes. Second-strand synthesis was carried out at 16 °C for 2 hours, followed by 85 °C for 20 minutes. Plates were snap-chilled, spun down at 2,000 r.c.f. for 1 minute and stored on ice before pooling. The protocol for pooling and in vitro transcription (IVT) was the same as SORT-seq^27^.

### VASA-drop: design of the droplet generation device

The droplet generation device for compressible barcoded bead and single-cell co-encapsulation (Extended Data Fig. 1c) was modified from previous designs^5,59^. The flow-focusing junction (80 µm) was narrowed to generate smaller droplets (0.55 nl) at high throughput (115 Hz).

### VASA-drop: design of droplet picoinjection devices

The design of both droplet picoinjector devices is based on the findings of a previous study^28^. Several key features were added to the architecture of previous designs to ameliorate the robustness of the injections in large droplets containing compressible barcoded beads:

1. Emulsion-diluting oil inlet, number 2 (Extended Data Fig. 1d), which reduces the packing of the emulsion to eliminate fragmentation of densely packed droplets before being re-injected in the picoinjection channel. This design feature allows for packed droplets to arrange into an evenly spaced monolayer that reduces fluctuations in volume of droplets after picoinjection.
2. Smooth narrowing of the reinjection chamber facilitating the ordering of droplets before spacing, which reduced droplet break-up.
3. Deepening of the outlet junction before the outlet, number 5 (Extended Data Fig. 1d,e, deep blue color), which stabilizes droplets and reduces droplet merging, which was observed during the rapid transition from the shallow microfluidic channel to a wide tubing or collection tip.

### VASA-drop: photolithography of microfluidic molds

The channel layout for the microfluidic chips was designed using AutoCAD (Autodesk) and printed out on a high-resolution film photomask (Micro Lithography Services). The designs in Extended Data Fig. 1 are deposited on https://openwetware.org/wiki/DropBase:Devices and can be found in the supplementary file ‘SI_VASAdrop CAD designs_5masks.dxf’. The microfluidic devices were fabricated following standard hard and soft lithography protocols that can be performed in local cleanrooms or outsourced to contract manufacturing companies. First, microfluidic molds were patterned on a 3-inch silicon wafer (MicroChemicals) using high-resolution film masks (Micro Lithography Services) and SU-8 2075 photoresists (Kayaku Advanced Materials). An MJB4 mask aligner (SÜSS MicroTec) was used to UV expose all the SU-8 spin-coated wafers. The thickness of the structures (corresponding to the depth of channels in the final microfluidic devices) was measured using a DektakXT Stylus profilometer (Bruker).

We used the following settings for photolithography:

**Table:** 

	Fabrication step (no. of layers)	
	1st layer	2nd layer (used for picoinjectors only)
Nominal thickness	80 µm	80 µm, 2nd layer (160-µm total thickness)
Resist used	SU-8 2075	SU-8 2075
Spin-coating speed	1st step: 10 s, 500 r.p.m.	1st step: 10 s, 500 r.p.m.
	2nd step: 30 s, 2,750 r.p.m.	2nd step: 30 s, 2,750 r.p.m.
Pre-baking	3 min at 65 ^o^C	3 min at 65 ^o^C
	9 min at 95 ^o^C	9 min at 95 ^o^C
Exposure (at ~10 mW cm^2^)	2× 10 s	2× 10 s
Post-baking	2 min at 65 ^o^C	2 min at 65 ^o^C
	7 min at 95 ^o^C	7 min at 95 ^o^C
Development in the beaker filled with 30–50 ml of PGMEA (propylene glycol methyl ether acetate, Sigma-Aldrich)	Approximately 5 minutes until all uncured SU-8 is removed from the wafer; development time depends on the intensity of manual agitation. The development step after 1st deposition is performed only for a 1-layer chip.	Approximately 10 minutes until all uncured SU-8 is removed from the wafer; development time depends on the intensity of manual agitation.
Hard baking (optional)	10 min at 200 ^o^C (only for a 1-layer chip)	10 min at 200 ^o^C
Measured range of thicknesses	80–84 µm	168–178 µm (second layer is usually ~20% thicker than nominal)

### VASA-drop: soft lithography

To manufacture PDMS microfluidic devices, 20–30 g of silicone elastomer base and curing agent (Sylgard 184, Dow Corning) were mixed at a 10:1 (w/w) ratio in a plastic cup and de-gassed in a vacuum chamber for 30 minutes. PDMS was then poured on a master wafer with SU-8 structures and cured in the oven at 65 °C for at least 4 hours. Next, the inlet holes were punched using two types of biopsy punchers with plungers (Kai Medical Laboratory): a 1.5-mm-diameter punch was used to make the inlet for the cell delivery tip, number 2 (Extended Data Fig. 1c); outlet for droplet collection tip, inlet number 5 (Extended Data Fig. 1c,d); and the inlets for droplet reinjection, number 1 (Extended Data Fig. 1d,e); whereas other inlets were made using a 1-mm-wide biopsy puncher. The patterned PDMS chip was then plasma bonded to a 52 mm × 76 mm × 1 mm (length × width × thickness) glass slide (VWR) in a low-pressure oxygen plasma generator (Femto, Diener Electronics). Next, the hydrophobic modification of microfluidic channels was performed by flushing the device with 1% (v/v) trichloro(1H,1H,2H,2H-perfluorooctyl)silane (Sigma-Aldrich) in HFE-7500 (3M) and baked on a hot plate at 75 °C for at least 30 minutes to evaporate the fluorocarbon oil and silane mix.

Although we have not used these commercial suppliers, we propose the following list of contract manufacturers for users who may not have access to photolithography/soft lithography: Fivephoton Biochemicals, Darwin Microfluidics, uFluidix, Flowjem and Microfactory.

### VASA-drop: cell loading and droplet collection/re-injection chamber manufacturing

#### Cell injection chamber

The cells were loaded in a cell loading tip pre-filled with mineral oil (Sigma-Aldrich). To manufacture the cell loading tip, a low-retention pipette tip with 200-µl volume capacity (Axygen) was cut at the top, in parallel to the rim and under the filter. A solidified 3-mm-thick piece of PDMS (Dow Corning) was punched from a slab of PDMS with a 5.0-mm sampling tool (EMS-Core). The circular piece of PDMS was then biopsy-punched with a 1-mm-wide biopsy puncher (Kai Medical Laboratory) in the middle. The circular piece of PDMS was pushed inside the tip while remaining parallel to the upper rim of the tip. A 1-ml glass syringe (SGE) was then pre-filled with 1 ml of mineral oil and connected to a 30-cm-long tubing (Portex, Smiths Medical) that can be inserted to a hole in the middle of the circular PDMS piece in the tip. Next, the tip was pre-filled with mineral oil by manually pushing the syringe, and the cell-containing solution was further aspirated with care as to not introduce any air bubble in the system. The tip can then be connected to the cell-encapsulation PDMS device, inlet number 2 (Extended Data Fig. 1c,g), and injection rates are modulated by a Nemesys syringe pump (Cetoni).

#### Droplet collection and reinjection chamber

A second type of tip chamber was designed to collect, incubate and re-inject droplets for each microfluidic step. To this end, a 5-mm-thick PDMS piece was punched from a slab of PDMS with an 8.0-mm sampling tool (EMS-Core) and re-punched in its center using a 1-mm-wide biopsy puncher (Kai Medical Laboratory), and a 30-cm-long tubing (Portex, Smiths Medical) was connected to the latter punched hole. The resulting piece of PDMS was then inserted into a 1-ml filterless pipette tip (Sigma-Aldrich) with a parallel orientation to the rim. Unsolidified PDMS (Dow Corning, 1:10 (w/w) ratio, de-gassed) was then deposited into the space between the rim and the circular PDMS piece at the top. The tip was then incubated at 65 °C for at least 4 hours and connected to a 1-ml glass syringe (SGE) pre-filled with mineral oil. The tip was then pre-filled with mineral oil by manually pushing the connected syringe. To collect the droplets after the initial encapsulation or at the end of the first picoinjection, the tip can be connected to the outlet of the devices, inlet number 5 (Extended Data Fig. 1c,d), and the syringe is disconnected to allow the evacuation of mineral oil as the tip gets loaded. For each of the two droplet picoinjection steps, the mineral oil can be pushed using a Nemesys syringe pump (Cetoni) to re-inject droplets into the picoinjectors, inlet number 1 (Extended Data Fig. 1d,e). For each of the re-injection and collection steps, the PDMS-punched holes on the microfluidic device need to be primed with 5% (w/w) 008-FluoroSurfactant (RAN Biotechnologies) in HFE-7500 (3M) to avoid a trapped air bubble to perturbate the stability of re-injection or the integrity of the emulsions. After droplet collection during the encapsulation and first picoinjection, the tip can be closed by inserting the narrower end of the tip into a glass-bonded PDMS plug, which closes the system and allows for incubation of the tip in the water bath (Extended Data Fig. 1f). The glass-bonded PDMS plug was fabricated before the experiment by punching a 8-mm-thick piece of PDMS with a 1.5-mm biopsy puncher that was then bonded to microscopy glass using an oxygen plasma.

### VASA-drop: microfluidic device operation

#### Polyacrylamide beads manufacturing

Barcoded polyacrylamide beads were manufactured following a previously described protocol^59^. In brief, a polyacrylamide mix was used to generate 60 µm of water-in-oil emulsions using a single-inlet flow-focusing device and collected in a 1.5-ml LoBind tube (Eppendorf) containing 200 µl of mineral oil (Sigma-Aldrich). The droplets were solidified overnight at 65 °C, de-emulsified using a 20% 1H,1H,2H,2H-perfluoro-1-octanol (Alfa Aesar) in HFE-7500 (3M) solution and stored at 4 °C for up to 6 months.

#### Co-encapsulation of cells and barcoded beads

A detailed protocol^59^ was used as a reference for droplet generation. First, the microfluidic droplet generation chip was installed on the stage of an inverted microscope (Olympus XI73). Next, two pieces of polyethylene tubing (Portex, Smiths Medical) were connected to two 1-ml gas-tight syringes (SGE) and filled with PBS (Lonza). The tubing was manually filled with PBS, and a small, 1-cm-long air bubble was left at the end tip of each tubing. The bead suspension and lysis mix were manually aspirated to the tubing, and the small air bubble provided a separation between the reagents and the PBS buffer. Then, 150 µl of cell suspension was manually aspirated into the cell loading tip pre-filled with mineral oil (Sigma-Aldrich). A fourth 2.5-ml glass syringe (SGE) was filled with 5% (w/w) 008-FluoroSurfactant (RAN Biotechnologies) in HFE-7500 (3M). Next, all three tubings and the cell chamber with cell suspension were inserted to the corresponding inlets of the droplet generation chip (Extended Data Fig. 1c). Four Nemesys syringe pumps (Cetoni) were used to flow each component, and the droplet formation was monitored using ×4 or ×10 objectives (Olympus) and a fast camera (Phantom Miro eX4) connected to the inverted microscope. After the device was primed and droplet generation was stabilized, the collection chamber was connected to the outlet.

#### Microfluidic device operation—picoinjection

Before starting the picoinjection of droplets containing single-cell lysates, the electrode sections, numbers 6 and 7 (Extended Data Fig. 1d,e) of the devices, were pre-filled with filtered 5 M NaCl as previously described^60^. The picoinjection chip was filled with 5% (w/w) 008-FluoroSurfactant (RAN Biotechnologies) in HFE-7500 (3M) using a pre-filled 2.5-ml glass syringe (SGE) connected to a piece of tubing (Portex, Smiths Medical). The reaction mix was primed, and the tip containing the emulsions (with fluorinated oil evacuated by pushing the glass syringe until the emulsions reached the exit of the tip) was primed and connected to the device. Next, flows of droplet emulsions, the reaction mix, the emulsion-diluting oil, number 2 (Extended Data Fig. 1d,e), and the droplet-spacing oil, number 3 (Extended Data Fig. 1d,e) were applied using the Nemesys syringe pumps (Cetoni). The droplets were diluted in a first instance in the re-injection chamber and then spaced with the second stream of oil in a flow-focusing re-injection junction. 5% (w/w) 008-FluoroSurfactant (RAN Biotechnologies) in HFE-7500 (3 M) was used for both diluting and spacing of droplets. The function generator (AIM & Thurlby Thandar Instruments) was set to generate square waves of 2.5V amplitude and 10kHz frequency, which were further amplified 100 times to 250 V by a Trek 601C-1 amplifier, which enabled coalescence-activated injection of the reagent into the droplets. The droplets were collected in a 1-ml collection tip connected at the outlet, number 5 (Extended Data Fig. 1d,e).

### VASA-drop: polyacrylamide bead barcoding

The bead barcoding procedure was performed as previously described^59^ with the inDrop v3 barcoding scheme^61^. In brief, the solidified barcoded beads were filtered and dispensed in four 96-well plates containing the first barcode from the inDrop v3 design, and the bead-bound adapter was extended using a Bst 2.0 DNA polymerase (NEB) after annealing the barcoded oligonucleotides. The reaction was then stopped, and the second strand was removed using a sodium hydroxide treatment. The second barcode was added in a similar fashion, and the beads were stored for up to 6 months at 4 °C.

### VASA-drop: cell encapsulation in water-in-oil emulsions

For the cultured cells and the embryos, we used a loading concentration of 450 cells per µl in 1× PBS (Lonza) with 15% OptiPrep (Sigma-Aldrich). The lysis mix was made fresh before each encapsulation, as follows: 0.5 mM dNTPs each (Thermo Fisher Scientific), 0.52% IGEPAL-CA630 (Sigma-Aldrich), 40 mM UltraPure Tris-HCl pH 8 (Life Technologies), 3.76× First-Strand Buffer (Invitrogen), 3 mM magnesium chloride (Ambion) and 6 U ml^−1^ of Thermolabile Proteinase K (NEB). The barcoded PAAm beads were prepared for encapsulation as previously described^5^. The lysis mix and bead suspensions were loaded in the tubing of two individual 1-ml SGE glass syringes filled with PBS (Lonza) and separated by an air bubble from the reagents in the tubing. The cells were loaded into a cell injection container pre-filled with mineral oil (Sigma-Aldrich). The injection flow rates for the droplet encapsulation device (Extended Data Fig. 1c) were as follows: the cell suspension was flown at 85 µl per hour, number 2 (Extended Data Fig. 1c); the bead suspension was flown at 65 µl per hour, number 3 (Extended Data Fig. 1c); the lysis solution was flown at 75 µl per hour, number 1 (Extended Data Fig. 1c); and 5% (w/w) 008-FluoroSurfactant (RAN Biotechnologies) in HFE-7500 (3M) was flown at 450 µl per hour using a 2.5-ml glass syringe (SGE), number 4 (Extended Data Fig. 1c). All flow rates for each microfluidic manipulation were controlled using Nemesys pumps (Cetoni). The average droplet size was ~0.55 nl for these flow rates and a microfluidic device depth of 80 µm. The droplets were collected for approximately 1 hour in a 1-ml pipette tip (Greiner) pre-filled with mineral oil at the outlet, number 5 (Extended Data Fig. 1c), and connected to a tubing via a PDMS connector (Extended Data Fig. 1g). The collection tip was then closed by connecting a 1-ml SGE glass syringe pre-filled with mineral oil to the tubing and the tip was then connected to a glass-bonded PDMS plug (Extended Data Fig. 1f).

### VASA-drop: cell lysis and RNA fragmentation

The tip container was further left at room temperature (23 °C) for 20 minutes to allow for cell lysis to occur, and the tip was then placed under a High-Intensity UV Inspection Lamp (UVP) that was switched on for 7 minutes for barcode photocleavage (Extended Data Fig. 1h). The container was then submerged in a water bath (Grant JB) placed at 85 °C for 6 minutes and 30 seconds. After incubation, the container was immediately submerged in an ice bucket filled up with half proportions of ice and water.

### VASA-drop: first picoinjection for RNA repair and poly(A) tailing

The droplets were re-injected in the first picoinjector device with the shorter re-injection channel (Extended Data Fig. 1d) to perform coalescence-induced merging with a poly(A) solution consisting of 26.6 mM Tris-HCl, pH 8 (Invitrogen), 15.8 mM DTT (Invitrogen), 0.83× First-Strand Buffer (Invitrogen), 0.19 mM ATP (NEB), 3.15 kU ml^−1^ of T4 Polynucleotide Kinase (NEB), 250 U ml^−1^ of *E. coli* poly(A) polymerase and 2.6 kU ml^−1^ of RNaseOUT (Applied Biosystems). The merging was applied by pre-filling the electrode section, numbers 6 and 7 (Extended Data Fig. 1d), of the device with 5 M NaCl, as previously described^60^. The flow rates used were 200 µl per hour for the droplet emulsion, number 1 (Extended Data Fig. 1d); 60 µl per hour for the poly(A) mix, number 4 (Extended Data Fig. 1d); 50 µl per hour for the emulsion-diluting oil, number 2 (Extended Data Fig. 1c); and 400 µl per hour for the droplet-spacing oil, number 3 (Extended Data Fig. 1d). This generated ~0.8 nl of droplets at 70 Hz. The droplets were collected in a 1-ml collection tip (Greiner) pre-filled with mineral oil and inserted to the outlet, number 5 (Extended Data Fig. 1d). At the end of the picoinjection, the collection tip was closed by connecting a 1-ml glass syringe (SGE) pre-filled with mineral oil (Sigma-Aldrich) to the tubing and connecting the narrower end of the tip to the glass-bonded PDMS plug. The tip container was then incubated for 25 minutes at room temperature (23 °C) and 8 minutes at 37 °C in a water bath (Grant JB) and then submerged in an ice-cold water bath for 2 minutes. The droplets were then processed for the second picoinjection.

### VASA-drop: second picoinjection for reverse transcription

The droplets were re-injected in the second picoinjector (Extended Data Fig. 1e) similarly to the previous step, although this time the droplets were collected in fractions of ~1,000 cells (~27 µl of loaded droplets) in 1-ml LoBind tubes (Eppendorf) pre-filled with 200 µl of mineral oil. The droplets were injected with a reverse transcription mix constituted of 25 mM Tris-HCl, pH 8 (Invitrogen), 8 mM DTT (Invitrogen), 0.75× First-Strand Buffer (Invitrogen), 1 mM dNTPs, 20 kU ml^−1^ of SuperScript III (Invitrogen) and 1.2 kU ml^−1^ of RNAseOUT (Applied Biosystems). The flow rates for the second picoinjection were as follows: 70 µl per hour for the emulsion-diluting oil, number 2 (Extended Data Fig. 1e); 700 µl per hour for the droplet-spacing oil, number 3 (Extended Data Fig. 1e); 300 µl per hour for the re-injected droplets, number 1 (Extended Data Fig. 1e); and 255 µl per hour for the reverse transcription mix, number 4 (Extended Data Fig. 1e). The collected fractions were incubated at 50 °C for 2 hours and then heat-inactivated at 70 °C for 20 minutes. For de-emulsification of the droplets, the mineral oil and the excessive fluorocarbon oil phase were aspirated and discarded. Then, 200 µl of filtered HFE-7500 was added to the emulsions, followed by 200 µl of 100% 1H,1H,2H,2H-perfluoro-1-octanol. The tubes were centrifuged for 5 seconds on a tabletop centrifuge, and then 300 µl of the oil phase was removed and 100 µl of fresh HFE-7500 oil was added, as well as 50 µl of TE buffer (Zymo). At this point, the fractions were stored at −80 °C. The protocol, up to and including the IVT step, was the same as for inDrop^59^.

### VASA-plate and VASA-drop: downstream library preparation and sequencing

For VASA-plate: after IVT, 2 µl of ExoSAP-IT (Applied Biosystems) was added, and each sample was incubated at 37 °C for 15 minutes. For both VASA-plate and VASA-drop: a 1.8× volumetric ratio AMPure XP clean-up was then performed, and the amplified RNA (aRNA) was eluted in 10 µl of nuclease-free water. The purified aRNA concentration was measured using a Qubit (Invitrogen), and the concentration was adjusted to a maximum of 100 ng µl^−1^. Next, 6 µl per sample was mixed with 2 µl of rRNA depletion probes (25 µM) (reverse complement of published probes^62^) and 2 µl of hybridization buffer (pH 7.5, 500 mM Tris-HCl, 1 M NaCl). Samples were incubated at 95 °C for 2 minutes and brought to 45 °C with a gradient of 0.1 °C per second. Once the probes were hybridized, 2 µl of Thermostable RNAseH (Epicentre) and 8 µl of RNAseH buffer (pH 7.5, 125 mM Tris-HCl, 250 mM NaCl, 50 mM MgCl_2_) was added. The reaction was incubated at 45 °C for 30 minutes and further kept on ice. Next, 4 µl of RQ DNAse I (Promega), 21 µl of nuclease-free water and 5 µl of CaCl_2_ (10 mM) were added to the reaction mixture. The mixture was further incubated at 37 °C for 30 minutes, followed by snap-cooling on ice. A 1.6× volumetric ratio AMPure XP clean-up was then performed, and the aRNA was eluted in 6 µl of nuclease-free water. Next, 1 µl of RA3 ligation oligonucleotide (20 µM; Supplementary Table 12) was added to 5 µl of the aRNA, and the reaction was brought to 70 °C for 2 minutes, followed by snap-cooling on ice. This was followed by the addition of 1 µl of 10× T4 RNA ligase reaction buffer (NEB), 1 µl of NEB T4 RNA Ligase2, truncated (NEB), 1 µl of RNAseOUT (Invitrogen) and 1 µl of nuclease-free water, The reaction was incubated at 25 °C for 1 hour, followed by snap-cooling on ice. The adapter-ligated aRNA was then mixed with 1 µl of dNTPs (10 mM each) (Promega) and 2 µl of RTP oligonucleotide (20 µM; Supplementary Table 12). The mixture was incubated at 65 °C for 5 minutes, followed by snap-cooling on ice. Next, 4 µl of 5× First-Strand Synthesis Buffer (Invitrogen), 1 µl of nuclease-free water, 1 µl of 0.1 M DTT (Invitrogen), 1 µl of RNAseOUT and 1 µl of SuperScript III were added to the sample. The reaction was incubated at 50 °C for 1 hour, followed by 70 °C for 15 minutes and then snap-cooled on ice. To reduce excess RNA material, 1 µl of RNAseA (Thermo Fisher Scientific) was further added to each tube, and the cDNA was incubated at 37 °C for 30 minutes, followed by a 1× volumetric AMPure XP clean-up. The cDNA was eluted in 20 µl of nuclease-free water. Half the material was used for the final PCR (10 µl). Each sample was mixed with 25 µl of NEBNext High-Fidelity 2× PCR Master Mix (VASA-plate) or Kapa HiFi HotStart PCR Mix (VASA-drop), 4 µl of PE1/PE2 primer mix (5 μM each)^1,27^ (VASA-plate) or 5 µl PE1/PE2 primer mix (5 μM each) (Supplementary Table 12) (VASA-drop) and 11 µl (VASA-plate) or 10 µl (VASA-drop) of nuclease-free water. The samples were amplified with the following PCR programs. VASA-plate: initial heat denaturation for 30 seconds at 98 °C, 7–8 cycles for 10 seconds at 98 °C, 30 seconds at 60 °C, 30 seconds at 72 °C and final extension for 10 minutes at 72 °C. VASA-drop: initial heat denaturation for 2 minutes at 98 °C, two cycles for 20 seconds at 98 °C, 30 seconds at 55 °C, 40 seconds at 72 °C, 5–6 cycles for 20 seconds at 98 °C, 30 seconds at 65 °C, 40 seconds at 72 °C and final extension for 5 minutes at 72 °C. Each amplified and indexed sample was purified twice using a 0.8× volumetric ratio of AMPure XP beads and eluted in 10 µl. Final libraries were checked for proper length on a Bioanalyzer (Agilent), and concentration was measured with a Qubit (Invitrogen). A detailed catalog of reagents and instrumentation is provided in Supplementary Table 14.

The VASA-drop samples were sequenced on a NovaSeq 6000 S2, 300 cycles flow cell (Illumina), with the following parameters: Read1 247 cycles, Index1 31 cycles, Index2 8 cycles, Read2 14 cycles. VASA-plate samples were sequenced on a NextSeq 500, high-output 150 cycles flow cell (Illumina), with the following parameters: Read1 26 cycles, Index 8 cycles, Read2 135 cycles.

### FASTQ file pre-processing in VASA-drop and 10x Chromium

Raw reads for VASA-drop were pre-processed with a Python script to have a favorable format for the pipeline (four reads were demultiplexed and rearranged into two reads). For each Read1, the UMI (6 nucleotides (nt) long in VASA-seq, 10 nt long in 10x Chromium) and the cell-specific barcode (16-nt long in VASA-seq, 14-nt long in 10x Chromium) were extracted. To determine the number of cells in each sample, first the total number of raw reads was determined for each possible barcode. Next, we plotted the histogram of log_10_(read number) for each possible barcode, which we fitted to a polynomial function that shows two or three minima. We used the position of the minimum with the highest value of log_10_(reads) as the threshold: only barcodes with reads above this threshold were used for downstream analysis. We merged sequenced barcodes that can be uniquely assigned to an accepted barcode with a Hamming distance of 2 nt or less.

### FASTQ file pre-processing in VASA-plate

Read1 starts with a 6-nt-long UFI/UMI, followed by an 8-nt-long cell-specific barcode. There are only 384 cell-specific barcodes, each one corresponding to a well in a 384-well plate (available in GSE176588). We merged sequenced barcodes that can be uniquely assigned to an accepted barcode with a Hamming distance of 1 nt or less.

### Mapping data (VASA-seq, 10x Chromium and Smart-seq v3)

Read2 was assigned to accepted barcodes (extracted from Read1) and trimmed with TrimGalore (version 0.4.3) with default parameters. Next, homopolymers at the end of the read were removed with cutadapt (version 2.10)^63^.

In silico ribosomal depletion was performed by mapping the trimmed reads to mouse or human rRNA (National Center for Biotechnology Information) using bwa mem and bwa aln (version 0.7.10)^64^. Multi-mappers and single-mappers were filtered out. The remaining reads were mapped to the mouse GRCm38 genome (Ensembl 99) or to the human GRCh38 genome (Ensembl 99) using STAR^65^ with default parameters. Assignment of reads to gene biotypes was performed according to the following hierarchy:

- All mappings falling in TEC transcripts were discarded.
- Reads fully falling inside a region annotated as miscRNA, mtRNA, mttRNA, TrJGene, miRNA, rRNA, ribozymes, sRNA, scaRNA, snRNA or snoRNA (for example, biotypes that do not have annotated introns) were assigned to such regions.
- When a read maps to multiple genes simultaneously (because of annotation overlap in the reference GTF file), exonic annotations were given preference to introns. In case all references are exonic or intronic, the read is assigned to a gene whose name is the sequence of all the target gene names.
- Reads falling into exon–intron junctions or inside introns are assigned to unspliced transcripts. Reads falling inside exonic regions are assigned to spliced transcripts.
- If at least one UFI of the same cell from the same transcript has been assigned to an unspliced transcript (because it is mapped in an intron or an intron–exon junction), all the other reads with the same UFI of the same cell for the same transcript are automatically assigned to unspliced transcripts even if they mapped to exons exclusively.

### Benchmarking against other methods

To determine the number of potential doublets, barcodes with more that 75% of the genes assigned to only one of either mouse or human were considered singlets. Cells with fewer than 7,500 UFIs were filtered out and not assigned to any organism. For gene body coverage, the BAM files for all single cells were used as a bulk. QoRTs^66^ was used to calculate coverage, and only protein-coding genes were kept. For Smart-seq3, both reads containing a UMI (5′ reads) and non-UMI-containing reads were used together. Average coverages were used for the plotting. To determine percentages of different biotypes, all single cells were used as a bulk. UMI/UFI filtering was carried out for reads where this was possible. For Smart-seq3, both reads containing a UMI (5′ reads) and non-UMI-containing reads were used together. For the gene detection assay, only cells that had been sequenced to the highest numbers of reads (reads with proper barcode and quality/homopolymers trimming) were used (75,000 for saturation curve and 750,000 deep sequencing comparison) (Extended Data Fig. 2f). For Smart-seq3, four cells, with much lower reads than the rest, were removed as they were considered failed libraries. Downsampling was carried out with DropletUtils^67^ on the count matrices (non-UMI/UFI filtered), based on the number of input reads and target reads, and only uniquely assigned genes were counted. For the percentage of intronic reads, each cell was used individually. UMI/UFI filtering was carried out for reads where this was possible. For Smart-seq3, both reads containing a UMI (5′ reads) and non-UMI-containing reads were used together. Mean and standard deviation were calculated and plotted.

### scRNA seq analysis for mouse VASA-seq libraries and individual timepoints

The Scrublet^68^ and Scanpy^69^ packages were used together with custom-made code. In brief, for VASA-seq, only cells with more than 10^4^ (E6.5), 10^3.5^ (E7.5, E8.5) and 10^3^ (E9.5) reads and fewer than 10^6^ transcripts were kept. Next, only cells in which 85–95% of transcripts belonging to protein-coding genes, 1–3% of transcripts belonging to lncRNA and 5–15% of transcripts belonging to small RNA were kept. Unspliced and spliced protein-coding genes were treated as different entries in our count tables to recover extra granularity in the downstream two-dimensional projection. Potential doublets as detected by Scrublet with default parameters were removed. The resulting count tables were library-size normalized to 10^4^ transcripts, and data were log-transformed with a pseudo-count equal to 1. Cells with a total transcript count to histone genes above 35 were assumed to be in S-phase (Fig. 3). Differential gene expression analysis between cells in S-phase and not S-phase was performed using the *t*-test to determine cell cycle genes (default scanpy.tl.rank_genes_groups function in Scanpy), for separate timepoints and all data together (Supplementary Table 4). Next, highly variable genes with mean log expression between 0.0125 and 5 were selected, and cell cycle genes were excluded. Number of counts and cell cycle properties were regressed out (Scanpy function scanpy.pp.regress.out), and data were z-transformed (scanpy.pp.scale). For all timepoints, we selected the top 50 principal components (except for E6.5, for which we selected the first 20). For each timepoint, we constructed a directed graph connecting nearest neighbor cells in the reduced principal components analysis (PCA) space, using the Manhattan metric as previously described^32^. Initially, for each cell, we identified its ten nearest neighbors. An outgoing edge from cell *i* to cell *j* was kept if the distance *d*_*ij*_ was less than the mean + 1.5× s.d. among all the distances connecting ten nearest neighbors. Cells that were not connected to any other cell were filtered out. The directed graph was converted to an undirected graph, and a two-dimensional UMAP was obtained as previously described^70^. We clustered the data using the Leiden algorithm (scanpy.tl.leiden, resolution set to 1) and performed differential gene expression between Leiden clusters using the *t*-test (default scanpy.tl.rank_genes_groups).

### scRNA seq analysis for mouse 10x Chromium libraries and individual timepoints

10x data were analyzed similarly to the VASA-seq data. Here, we kept cells with more than 10^3.5^ and fewer than 10^6^ uniquely detected transcripts and with 85–97% protein-coding transcripts. Cell cycle genes were not removed from the set of highly variable genes, and cell cycle regression was not performed. The effect of the libraries was regressed out before Z-score scaling.

### Comparison between 10x Chromium and VASA-seq embryo data

For the comparison, only reads mapping at the 80% 3′ end of gene bodies were used to generate count tables for both VASA-seq and 10x Chromium. Only genes expressed in both technologies were used for the comparison. The technology and the number of counts were regressed out from the combined VASA–10x Chromium dataset, and dimensionality reduction was performed by PCA. Manhattan-based distances between cells were calculated in the combined PCA space. Equivalent clusters were defined by fist clustering each dataset for each timepoint independently. Second, for a given cluster and a reference technology (for example, VASA-seq), a background histogram of the distances between cells in that cluster and their corresponding first nearest neighbor in the target technology (for example, 10x Chromium) was obtained. Finally, each cell in the target technology was assigned to the cluster of its nearest neighbor in the reference technology. Cells with low transfer scores were excluded, and equivalent clusters with low numbers of cells in any technology were excluded from the downstream analysis. Equivalent clusters between VASA-seq and 10x Chromium were defined as groups of cells with identical 10x Chromium and VASA cluster assignments. To assign a germ layer to each equivalent cluster, published annotations for the 10x Chromium data^24^ were used (epiblast: epiblast, primitive streak, anterior primitive streak, caudal epiblast and NMP; ectoderm: ExE ectoderm, caudal neurectoderm, rostral neurectoderm, surface ectoderm, forebrain/midbrain/hindbrain, neural crest and spinal cord; mesoderm: nascent mesoderm, caudal mesoderm, ExE mesoderm, intermediate mesoderm, mesenchyme, mixed mesoderm, paraxial mesoderm, pharyngeal mesoderm, somitic mesoderm and cardiomyocytes; endoderm: allantois, def. endoderm, ExE endoderm, gut, parietal endoderm and visceral endoderm; blood: blood progenitors 1, blood progenitors 2, erythroid1, hematoendothelial progenitors, endothelium, erythroid2 and erythroid3; and PGC: PGC). The prevalent annotation for each equivalent cluster was used.

### Master UMAP for VASA-drop mouse embryo data

The master UMAP, where all cells for all timepoints are integrated together, was obtained as previously described^32^. In brief, we first built a directed graph. For each cell in each timepoint, we found the top 30 nearest neighbors in the subset of cells from the same timepoint and the previous timepoint (cells from E6.5 are only connected to cells from E6.5). To do so, all the cells in the subset are projected to the PCA space of the latest timepoint, and distances are calculated using the Manhattan metric. Next, the undirected graph was extracted and used to project the data to the two-dimensional UMAP.

### Expanding the transcriptome annotation

A total of 33,662 demultiplexed and ribo-depleted FASTQ files for each cell were used to reconstruct the transcriptome and quantify AS events. To this end, we implemented a custom computational workflow using Snakemake^71^ based on Hisat2/StringTie2 (ref. ^72^) and additional custom scripts. First, PCR duplicates were removed through a custom Python script that calculates pairwise identity across UMIs for each sequenced read within single cells. Then, reads were grouped by previously obtained Leiden clusters and mapped to the reference mouse genome assembly, version GRCm38, using HISAT2 (ref. ^73^). We performed the alignments implementing the recommended configuration for HISAT2 and genome indexing to ensure an optimal performance during later steps of the transcriptome assembly^74^.

The alignments for each cluster were assembled and then merged using StringTie2 (ref. ^72^). The resulting GTF file was then compared to the input transcriptome annotation using gtfcompare^72^, which assigns a classification code to each assembled transcript, which is subsequently used to filter transcripts with codes that indicate additional portions of annotated transcripts or novel genes. Novel transcripts spanning three or more exons that were classified under code k, m, n, j, x, i or y were appended to the input transcriptome annotation, expanding the original set of annotated transcripts. Finally, to further improve the quality of potentially novel transcripts, additional custom filtering steps were implemented to avoid novel transcripts due to false-positive novel exons. This filter is particularly important for transcripts assembled from reads that are mapped to repetitive sequences or exons that are ≤30 nt, which can arise from HISAT2 misalignments. To annotate potentially novel microexons, we used MicroExonator, a specialized computational workflow for discovering and quantifying microexons^35^. After running MicroExonator’s discovery module, we obtained a transcriptome annotation, which was later processed with custom scripts to limit the number of alternative transcription start and end sites.

### Quantification of AS events across cell types

The final GTF from the expanded transcriptome annotation was used to quantify isoforms and AS events using Whippet^36^. We ran Whippet through MicroExonator’s downstream module to profile AS events using scRNA-seq data, which enabled randomized aggregations of cells into pseudo-bulks and pairwise comparisons of AS profiles across cell types. To determine relevant pairwise comparison of AS profiles across cell types, we used PAGA^37^ to calculate connectivities between cell clusters based on gene expression. We then compared the 72 pairs of clusters that have a connectivity ≥0.05. For each comparison, cells from each cluster were randomly pooled to form at least three different pseudo-bulks of 200 or fewer cells. To detect reproducible changes of splicing node inclusion across cell types, random pseudo-bulk pooling and differential inclusion steps were repeated 50 times for each pairwise comparison, avoiding the detection of spurious splicing events. As part of MicroExonator’s workflow, the obtained probabilities of each splicing node to be differentially included were used to fit a beta distribution model and calculate CDF-beta values for each event. DISNs were defined as events with CDF-beta values equal to or lower than 0.05. To identify SNMs, we calculated the average ψ values for each splicing node across three randomly defined pseudo-bulk samples for each cell cluster. For splicing nodes where ψ values could be quantified based on at least ten reads across at least 50 pseudo-bulks, we calculated the Z-score by comparing to all other pseudo-bulks. We considered a splicing node as an SNM for a given cell type if at least two pseudo-bulks had significant Z-scores (*P* ≤ 0.05) and an absolute difference of at least 0.3 from the mean across all pseudo-bulks. To show some functional consequences of detected AS events for protein function, we used the drawProteins package^75^ to draw scaled diagrams of protein domains and other features annotated in UniProt^76^.

### Reporting summary

Further information on research design is available in the Nature Research Reporting Summary linked to this article.

## Online content

Any methods, additional references, Nature Research reporting summaries, source data, extended data, supplementary information, acknowledgements, peer review information; details of author contributions and competing interests; and statements of data and code availability are available at 10.1038/s41587-022-01361-8.

## Supplementary information

Reporting Summary

Supplementary Table 1 Cell encapsulation efficiencies in VASA-drop calculated across triplicate experiments.

Supplementary Table 2 Lists of all equivalent cluster markers for protein-coding (spliced and unspliced) genes and for lncRNA for each technology at different timepoints, detected using the default function from the Scanpy package. The statistical test used was a one-sided *t*-test; corrected and uncorrected *P* values for multiple comparisons are provided.

Supplementary Table 3 List of differentially expressed genes as detected using the *t*-test (absolute value of the log_2_ fold change >4; *P* < 0.001) between 10x and VASA-seq for each equivalent cluster in each timepoint, showing their mean expression, the standard deviation and the fraction of cells expressing the genes within the cluster. A pseudo-count equal to the minimum mean expression value above zero was used to extract the log_2_ fold change. The statistical test used was a two-sided *t*-test, and *P* values are not corrected for multiple comparisons.

Supplementary Table 4 Cell cycle genes during mouse embryonic development, obtained by differential gene expression analysis between S-phase and non-S-phase cells, for either pooled or separate timepoints. The statistical test used was a two-sided *t*-test, and *P* values are not corrected for multiple comparisons.

Supplementary Table 5 Regressed data were clustered using the Leiden algorithm. Table showing differentially expressed genes per cluster. These clusters were further used for cell type calling based on the differentially expressed genes (marker genes). The statistical test used was a one-sided *t*-test, and *P* values are not corrected for multiple comparisons. Corrected values for multiple comparisons and uncorrected *P* values are provided.

Supplementary Table 6 Differentially expressed histone genes between Leiden clusters/cell types. Both uniquely and multi-assigned histone genes were included. The statistical test used was a two-sided *t*-test, and *P* values are not corrected for multiple comparisons.

Supplementary Table 7 Genes that contributed to the RNA velocity vector for VASA-seq and 10x. We found that most significant genes were shared between the methods (1,492), but VASA-seq detected a large number of additional genes (1,069).

Supplementary Table 8 List of differentially included nodes that were found across the different comparisons made between cell clusters. Leiden cluster IDs that were compared are indicated by A.cluster_names and B.cluster_names columns. Columns 4–8 provide the information corresponding to the assessed splicing nodes. Each comparison was repeated 50 times, and summary statistics (such as mean, standard deviation and variance) are reported for each splicing node that was differentially included across the computed comparisons between clusters. Finally, associated CDF-beta values to each listed splicing node are indicated on the CDF-beta column.

Supplementary Table 9 List of SNMs identified across cell clusters. For each splicing node, the coordinate and the Leiden ID of the cluster, where they were found to be markers, are indicated by the Coord and Leiden columns, respectively. Additional stats that were used to identify each SNM are reported between columns 4–9.

Supplementary Table 10 List of DISNs between the FHF and PHT, as identified by the MicroExonator pipeline. Positive DeltaPSI.mean values indicate inclusion in the FHF, whereas negative values indicate inclusion in the PHT.

Supplementary Table 11 List of DISNs between the primitive erythroids at E7.5 and E9.5, as identified by the MicroExonator pipeline. Positive DeltaPSI.mean values indicate inclusion at E7.5, whereas negative values indicate inclusion at E9.5. Abs_psi represents the absolute value of the differential PSI.

Supplementary Table 12 List of oligonucleotides not found in cited publications. 195 rRNA depletion probes, two oligos for library prep of VASA-drop samples and 92 dual index PCR primers for amplification of VASA-drop samples.

Supplementary Table 13 Cost calculations for the VASA-drop and VASA-plate methodologies compared to Smart-seq3 and 10x Genomics Chromium.

Supplementary Table 14 List of reagents and equipment for running the VASA-drop workflow.

Supplementary Video 1 High-throughput co-encapsulation of single cells, barcoded polyacrylamide beads and lysis/fragmentation mix in water-in-oil emulsions.

Supplementary Video 2 High-throughput injection of the polyA/repair (first picoinjection) or reverse transcription (second picoinjection) mixes in water-in-oil emulsions containing single-cell lysates and barcoded polyacrylamide beads.

Supplementary Data 1 Designs for the fabrication of the microfluidic devices for the VASA-drop protocol. The first design (top) represents the VASA-drop encapsulator microfluidic design (80 μm single layer). The second design (second row) represents the first picoinjector for injection of the polyA and repair mixes. The design is a two-layer design (80 μm each). The third design (third row) represents the second picoinjector for injection of the reverse transcriptase mix. The design is a two-layer design (80 μm each).

## Data Availability

Data are available at the Gene Expression Omnibus under accession number GSE176588. For benchmarking, we used the following accession numbers: E-MTAB-8735 (Smart-seq3) and GSE151334 (Smart-seq-total). We obtained the FASTQ files for HEK293T sequencing with 10x Genomics Chromium version 3.1 on their dataset page. For the murine atlas generated with 10x Genomics Chromium, we used accession number E-MTAB6967. We used the GRCh38 genome (Ensembl 99) as reference for sequencing data from human samples and GRCm38 genome (Ensembl 99) as reference for sequencing data from mouse samples.

## Code availability

Mapping and analysis scripts are available at https://github.com/hemberg-lab/VASAseq_2022.
