# Supplementary material for: High-throughput total RNA sequencing in single cells using VASA-seq
Source: Nat Biotechnol. 2022 Jun 27;40(12):1780–93. doi: 10.1038/s41587-022-01361-8 (PMC9750877; doi:10.1038/s41587-022-01361-8)
Supplement: Supplementary file 1 — Reporting Summary [file 41587_2022_1361_MOESM1_ESM.pdf]

nature portfolio

Corresponding author(s): Alexander van Oudenaarden
Florian Hollfelder

Last updated by author(s): 2022/05/10

Reporting Summary

Nature Portfolio wishes to improve the reproducibility of the work that we publish. This form provides structure for consistency and transparency in reporting. For further information on Nature Portfolio policies, see our Editorial Policies and the Editorial Policy Checklist.

Statistics

For all statistical analyses, confirm that the following items are present in the figure legend, table legend, main text, or Methods section.

n/a	Confirmed	
☐	☒	The exact sample size (*n*) for each experimental group/condition, given as a discrete number and unit of measurement
☐	☒	A statement on whether measurements were taken from distinct samples or whether the same sample was measured repeatedly
☐	☒	The statistical test(s) used AND whether they are one- or two-sided<br>*Only common tests should be described solely by name; describe more complex techniques in the Methods section.*
☐	☒	A description of all covariates tested
☐	☒	A description of any assumptions or corrections, such as tests of normality and adjustment for multiple comparisons
☐	☒	A full description of the statistical parameters including central tendency (e.g. means) or other basic estimates (e.g. regression coefficient) AND variation (e.g. standard deviation) or associated estimates of uncertainty (e.g. confidence intervals)
☐	☒	For null hypothesis testing, the test statistic (e.g. *F*, *t*, *r*) with confidence intervals, effect sizes, degrees of freedom and *P* value noted<br>*Give P values as exact values whenever suitable.*
☐	☒	For Bayesian analysis, information on the choice of priors and Markov chain Monte Carlo settings
☐	☒	For hierarchical and complex designs, identification of the appropriate level for tests and full reporting of outcomes
☒	☐	Estimates of effect sizes (e.g. Cohen's *d*, Pearson's *r*), indicating how they were calculated

Our web collection on statistics for biologists contains articles on many of the points above.

Software and code

Policy information about availability of computer code

Data collection	The VASA-drop samples were sequenced on a Novaseq 6000 S2, 300 cycles flow cell (Illumina), with the following parameters: Read1 247 cycles, Index1 31 cycles, Index 2 8 cycles, Read2 14 cycles. VASA-plate samples were sequenced on a Nextseq 500 High otput 150 cycles flow cell (Illumina), with the following parameters: Read1 26 cycles, Index 8 cycles, Read2 135 cycles.
Data analysis	Data was analyzed using the following packages for the analysis from Figure 1-4 Scanpy v1.5.1, Trim Galore v0.4.3, Cutadapt v2.10, STAR aligner v2.7.3a, bwa v0.7.10, scVelo v0.2.2, Samtools v1.3.1, bedtools v2.29.1, Scrublet v0.2.1, QoRTs v1.2.42 and dropletUtils v1.6.1. For the analysis in Figures 5 and 6, we used the following software: Whippet v1.5.1, MicroExonator v1.0.0, bwa v0.7.15, Hisat2 v2.1.4, Snakemake 5.32.0, bedtools v2.29.2, ggsashimi v0.5.0, GFF utilities v0.9, UpsetR v1.4.0, drawProteins v1.0.2, StringTie v2.1.4. Autocad 2018 was used for generating the microfluidic designs. Link to code: https://github.com/hemberg-lab/VASAseq_2022

For manuscripts utilizing custom algorithms or software that are central to the research but not yet described in published literature, software must be made available to editors and reviewers. We strongly encourage code deposition in a community repository (e.g. GitHub). See the Nature Portfolio guidelines for submitting code & software for further information.

Data

Policy information about availability of data

All manuscripts must include a data availability statement. This statement should provide the following information, where applicable:
- Accession codes, unique identifiers, or web links for publicly available datasets
- A description of any restrictions on data availability
- For clinical datasets or third party data, please ensure that the statement adheres to our policy

Data is available at GEO with accession number: GSE176588. For benchmarking, we used the following accession numbers: E-MTAB-8735 (Smart-seq3), GSE151334

(Smart-seq total); and obtained the fastq files for HEK293T sequencing qith 10x Genomics Chromium v3.1 on their dataset page.For the murine atlas generated with 10x Genomics Chromium, we used the accession number: E-MTAB6967. We used the GRCh38 genome (ensembl 99) as reference for sequencing data from human samples and GRCm38 genome (ensembl 99) as reference for sequencing data from mouse samples.

Field-specific reporting

Please select the one below that is the best fit for your research. If you are not sure, read the appropriate sections before making your selection.

☒ Life sciences ☐ Behavioural & social sciences ☐ Ecological, evolutionary & environmental sciences

For a reference copy of the document with all sections, see nature.com/documents/nr-reporting-summary-flat.pdf

Life sciences study design

All studies must disclose on these points even when the disclosure is negative.

Sample size	>30,000 cells sequenced from C57BL/6 embryos at stage E6.5, E7.5, E8.5 and E9.5. Sample size for each timepoint up to E8.5 was determined to match the equivalent dataset from Pijuan-Sala et al. (Nature, 2019). E9.5 sample size was estimated relatively to E8.5 sample size.
Data exclusions	Cellular doublets and low complexity cells were excluded from the analysis.
Replication	The datasets generated were benchmarked against equivalent technologies: Smart-seq3, Smart-seq total and 10x Genomics Chromium. Samples for VASA-drop were collected in batches of ~1,000 cells to underline the absence of batch effect.
Randomization	N/A
Blinding	N/A

Reporting for specific materials, systems and methods

We require information from authors about some types of materials, experimental systems and methods used in many studies. Here, indicate whether each material, system or method listed is relevant to your study. If you are not sure if a list item applies to your research, read the appropriate section before selecting a response.

Materials & experimental systems

n/a	Involved in the study	
☒	☐	Antibodies
☐	☒	Eukaryotic cell lines
☒	☐	Palaeontology and archaeology
☐	☒	Animals and other organisms
☒	☐	Human research participants
☒	☐	Clinical data
☒	☐	Dual use research of concern

Methods

n/a	Involved in the study	
☒	☐	ChIP-seq
☒	☐	Flow cytometry
☒	☐	MRI-based neuroimaging

Eukaryotic cell lines

Policy information about cell lines

Cell line source(s)	HEK293T cells were a generous gift from Prof. Marc de la Roche (Department of Biochemistry, University of Cambridge). Mouse embryonic stem cells E14Tg2a wild-type were a generous donation from Prof. Austin Smith. The C57BL/6 mouse embryos were obtained from Charles River or the Cambridge Stem Cell Institute.
Authentication	None of the cells were authenticated
Mycoplasma contamination	Cells were not screened for Mycoplasma contamination.
Commonly misidentified lines (See ICLAC register)	N/A

Animals and other organisms

Policy information about studies involving animals; ARRIVE guidelines recommended for reporting animal research

Laboratory animals	Mus musculus C57BL/6 males and females at stages E6.5, E7.5, E8.5 and E9.5.

Wild animals	N/A
Field-collected samples	N/A
Ethics oversight	Use of animals in this project was approved by the Animal Welfare and Ethical Review Body for the University of Cambridge and relevant Home Office licence PPL (76777883) is in place.

Note that full information on the approval of the study protocol must also be provided in the manuscript.
